# Behavioral addictions and their reciprocal associations with each other, substance use disorders, and mental health problems: Findings from a longitudinal cohort study of young Swiss men

**DOI:** 10.1556/2006.2025.00078

**Published:** 2025-09-15

**Authors:** Matthias Wicki, Joseph Studer, Simon Marmet, Yasser Khazaal, Gerhard Gmel

**Affiliations:** 1 Addiction Medicine, Lausanne University Hospital and University of Lausanne, Lausanne, Switzerland; 2 Institute for Research, Development and Evaluation, Bern University of Teacher Education, Bern, Switzerland; 3 Department of Psychiatry, North-West Vaud Adult Psychiatry Service, Lausanne University Hospital and University of Lausanne, Lausanne, Switzerland; 4 Addiction Switzerland, Lausanne, Switzerland; 5 Centre for Addiction and Mental Health, Toronto, Canada; 6 Alcohol and Health Research Unit, University of the West of England, Bristol, United Kingdom

**Keywords:** Cohort Study on Substance Use Risk Factors (C-SURF), behavioral addictions, substance use disorders, mental health, latent change score model, cross-domain coupling

## Abstract

**Background and Aims:**

The co-occurrence of behavioral addictions (BAs) and substance use disorders (SUDs) or other mental health problems (MHPs) is well documented. However, there is limited evidence on associations between changes in the severity of BAs, SUDs, and MHPs, or their directions of influence or causation.

**Methods:**

A non-self-selecting sample of 5,611 young Swiss men (mean age 25.5 at baseline and 28.3 at follow-up) completed a self-reporting questionnaire on various BAs (gambling, gaming, internet, internet pornography, smartphone, work), SUDs (alcohol, cannabis) and MHPs (major depressive disorder, ADHD, borderline personality disorder, social anxiety disorder). Latent change score models were used to evaluate pairwise, bidirectional associations in symptom severity among different BAs, and between BAs and SUDs or MHPs.

**Results:**

Overall, changes in each BA's symptom severity were significantly and positively correlated with changes in the symptom severity of other BAs, alcohol use disorder, and MHPs; for cannabis use disorder, such correlations were only found with gaming and work. Significant bidirectional cross-lagged associations were found between the severity of BAs and MHPs, and between the severity of internet and smartphone addiction and other BAs. For SUDs, cross-lagged pathways were often not significant (e.g., with gambling or pornography) or even negative (between cannabis use disorder and work).

**Discussion and Conclusions:**

This study provides strong evidence that BAs and MHPs mutually reinforce each other over time. While this interplay can develop and maintain dysfunction, it may also enable positive change, highlighting the need for a comprehensive theoretical framework and integrated intervention approaches.

## Introduction

Behavioral addictions (BAs) often co-occur with addictive behaviors and mental health problems (MHPs; Starcevic & Khazaal, 2017; Sussman, Lisha, & Griffiths, 2011). However, it remains unclear whether BAs lead to psychiatric disorders or vice versa, or how changes in BA symptoms severity relate to changes MHP symptoms severity (Kim et al., 2021; Sinclair et al., 2021; Starcevic & Khazaal, 2017). The present study, therefore, examined longitudinal associations and mutual effects between a broad range of BAs (gambling, gaming, internet, smartphone, pornography, work), between BAs and substance use disorders (SUDs; alcohol use disorder [AUD], cannabis use disorder [CUD]), and between BAs and MHPs (major depressive disorder [MDD], attention deficit hyperactivity disorder [ADHD], borderline personality disorder [BPD], social anxiety disorder [SAD]).

Debate persists on whether repetitive, problematic behaviors should be viewed as BAs or impulse control disorders, and whether everyday behaviors are being over-pathologized (Billieux, Schimmenti, Khazaal, Maurage, & Heeren, 2015; Brand et al., 2022; Griffiths, 2022; Kardefelt-Winther et al., 2017). The DSM-5 only recognizes gambling disorder as a BA, with internet gaming disorder needing further study (American Psychiatric Association [APA], 2013), whereas the ICD-11 classifies both gambling and gaming as addictive behaviors (World Health Organization, 2018). Nonetheless, due to negative consequences among some people, other potentially problematic behaviors such as internet use (Montag, Wegmann, Sariyska, Demetrovics, & Brand, 2021; Pontes, Satel, & McDowall, 2022), pornography use (Gola et al., 2022; Khazaal et al., 2019), smartphone use (Panova & Carbonell, 2018; Rozgonjuk, Montag, & Elhai, 2022), and work (Clark, Michel, Zhdanova, Pui, & Baltes, 2016; Griffiths, Demetrovics, & Atroszko, 2018) are also frequently considered BAs. While acknowledging the ongoing discourse, we use the term ‘BA’ as an umbrella term for gambling disorder, problematic gaming, problematic internet use, problematic internet pornography use, problematic smartphone use, and work addiction, as assessed with their respective self-report instruments (e.g., Bergen Work Addiction Scale; Andreassen, Griffiths, Hetland, & Pallesen, 2012).

### Co-occurrence of BAs with SUDs and MHPs

The co-occurrence of BAs with each other and with SUDs and MHPs, as reviewed by Sussman et al. (2011) and Starcevic and Khazaal (2017), has been consistently confirmed (e.g., Binnie & Reavey, 2020; Bisen & Deshpande, 2018; Gomez, Stavropoulos, Brown, & Griffiths, 2022; Griffiths et al., 2018; Hartmann & Blaszczynski, 2018; Männikkö, Ruotsalainen, Miettunen, Pontes, & Kääriäinen, 2020; Marmet et al., 2019; Ratan, Parrish, Zaman, Alotaibi, & Hosseinzadeh, 2021). However, while BAs and SUDs often co-occur, factor-analytic findings suggest they form separate but related clusters (Gomez et al., 2022).

The etiological implications of the co-occurrence of BAs, SUDs and MHPs have remained unclear, as most empirical studies were cross-sectional (Starcevic & Khazaal, 2017). Even a review of longitudinal studies found no clear conclusions whether BAs predispose individuals to MHPs or vice versa (Hartmann & Blaszczynski, 2018). However, a recent study on gambling (Kim, Tabri, & Hodgins, 2024) found a simultaneous decline in the severity of problematic gambling and other addictive behaviors among subclinical cases, while more severe cases remained stable in their problem severity.

### Explaining the development, maintenance, and co-occurrence of addictions

The Interaction of Person-Affect-Cognition-Execution (I-PACE) model (Brand et al., 2016, 2019, 2025) proposes that the development and maintenance of addictive behaviors are shaped by predisposing person-related factors, including psychopathological features (e.g., MDD, SAD, ADHD) and personality traits (e.g., low conscientiousness, impulsivity). These interact with moderators (e.g., coping style, cognitive biases) and mediators (e.g., craving, reduced inhibitory control). Over time, repeated behaviors form habits that become less gratifying and more compensatory. Initially developed for problematic internet use (Brand et al., 2016), I-PACE has been applied to other BAs, such as gaming, gambling, pornography use, smartphone use, and SUDs (Elhai, Yang, Dempsey, & Montag, 2020; Marino et al., 2023; Reichl, Enewoldsen, Müller, & Steins-Loeber, 2023; Xu, Gao, Lian, Chen, & Zhou, 2023; Zhou et al., 2018).

Two main scenarios may explain the co-occurrence of addictions and other MHPs (Lieb, 2014): (1) the conditions are causally related; (2) they share common risk factors. Longitudinal studies cannot establish causality but help evaluate these scenarios by examining correlated and directional change (Littlefield et al., 2009, 2012). Correlated change means changes in one condition are associated with changes in the other over time. Directional change means one conditions' baseline severity predicts change in the other. Scenario one expects correlated and directional changes; scenario two expects correlated but no directional change. The I-PACE model (Brand et al., 2025) accommodates both scenarios: scenario one through its feedback loops linking affect, cognition, and addictive behavior, and scenario two through shared characteristics like genetics or early childhood experiences.

### The present study

**Fig. 1.**
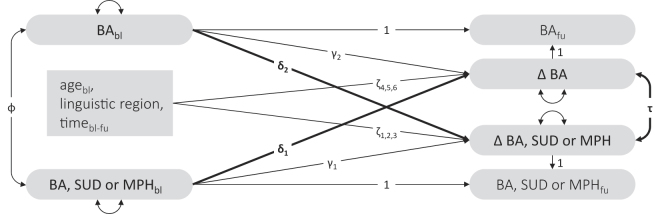
Latent change score model estimating correlated changes (τ) and directional changes (δ_1_, δ_2_) between behavioral addictions (BA) and one other BA, substance use disorder (SUD), or mental health problem (MHP) *Note*: The model was estimated separately for each BA (gambling, gaming, internet, pornography, smartphone, and work) in combination with one other BA, a SUD (alcohol use disorder, cannabis use disorder), or an MHP (major depressive disorder, ADHD, borderline personality disorder, and social anxiety disorder). Rounded grey shapes depict continuous, latent variables representing symptoms of BA, SUD or MHP. The grey rectangle represents observed variables (age_bl_, linguistic region, time_bl-fu_). Residuals and variances are illustrated as double-headed arrows entering a grey shape. Double-headed arrows (τ, Φ) represent correlations between two grey shapes, while single-headed arrows indicate paths (δ, γ, ζ) between them. Stability parameters (γ_1_, γ_2_) adjust for regression to the mean, whereas ζ adjusts for covariates. Covariances between age, language, duration, BA_bl_, and BA/SUD/MHP_bl_, as well as means, are estimated but not shown, for visual clarity.

The present study used latent change score (LCS) models with cross-domain coupling (Fig. 1) to examine reciprocal associations—correlated and directional changes—between BAs and between BAs and SUDs and MHPs. The analyses were based on a three-year follow-up study of a non-self-selecting general population sample of young Swiss men. The study employed a dimensional approach to addiction (Haslam, McGrath, Viechtbauer, & Kuppens, 2020; Volkow, 2020), focusing on the symptom severity rather than a taxonic (i.e., categorical) approach based on symptom count cutoffs.

There is limited knowledge regarding the bidirectional associations between BAs, and between BAs and SUDs and MHPs (Faelens et al., 2021; Marmet et al., 2019; Starcevic & Khazaal, 2017). Understanding whether directional or correlated changes occur between these conditions is crucial for determining whether cross-sectionally correlated variables are causally related or share a common causal factor. Addressing this knowledge gap is essential to improving theoretical models and informing effective prevention and intervention strategies (Brand et al., 2025; Dom & Moggi, 2014; Morisano, Babor, & Robaina, 2014; van Wamel, van Rooijen, & Kroon, 2014).

The present study's first aim was to examine correlated changes (τ; Fig. 1) between pairs of latent variables, specifically, whether changes in the symptom severity of BAs were correlated with changes in the symptom severity of other BAs, SUDs, and MHPs. Based on cross-sectional associations documented in the literature, we hypothesized that all examined correlated changes would be positive.

The second aim was to examine directional changes (δ; Fig. 1) between pairs of latent variables—specifically, whether the baseline symptom severity of BAs were associated with changes in the symptom severity of other BAs, SUDs, and MHPs over time, and vice versa. The I-PACE model (Brand et al., 2025) suggests these variables are connected to varying degrees: BAs and MHPs are directly linked by feedback loops (with MDD, ADHD, and SAD representing psychopathological features, and BPD reflecting personality traits such as impulsivity, low self-esteem, and low conscientiousness; Leichsenring et al., 2023; Samuel & Widiger, 2008); different BAs may be functionally equivalent or similar to one another and share affective or cognitive biases, common triggers, or predisposing variables; and BAs and SUDs often share MHPs or background factors like genetics or early experiences.

Based on the literature, we expected the most significant directional changes to be between BAs and MHPs, followed by those between BAs involving similar media or functions (e.g., internet, pornography, smartphones), and least between BAs and SUDs.

## Methods

### Sample

This study used data from the third and fourth waves of the Cohort Study on Substance Use Risk Factors (C-SURF), including assessments of various BAs (Gmel et al., 2015, 2021; Studer, Baggio, et al., 2013; Studer, Mohler-Kuo, et al., 2013). C-SURF sampled a cohort of young Swiss men representative of 21 of Switzerland's 26 cantons. Almost all Swiss men aged 19 must report to one of six recruitment centers to have their eligibility for mandatory military service assessed. All 13,237 young men who reported to Lausanne, Mels, and Windish between August 2010 and November 2011 were invited to participate in the future C-SURF survey, and 7,556 (57.1%) provided their written consent to do so. Recruitment centers were used solely to inform and enroll participants; all the C-SURF questionnaires were completed at home, and the army received no information on potential recruits' responses. Participation was rewarded with a voucher. Participants and non-participants were compared using data from a short army-administered questionnaire completed by over 94% of the source population, showing similar levels of education, urbanicity, and substance use (Studer, Baggio, et al., 2013). Further information on the C-SURF study and its data can be found online (Gmel et al., 2021) and in previous publications (Gmel et al., 2015; Studer, Baggio, et al., 2013; Studer, Mohler-Kuo, et al., 2013).

C-SURF's third wave survey took place between April 2016 and March 2018, with 5,516 respondents (73.0% response rate of those who had given informed consent at baseline). Fourth wave data were collected between April 2019 and September 2020, with 5,367 participants (94.7% retention from waves three to four:). To avoid bias related to the COVID-19 pandemic, we excluded 270 fourth-wave questionnaires (5.0%) answered after February 25, 2020, the date of Switzerland's first confirmed case of COVID-19 (Federal Office of Public Health, 2020). Thus, this study's analytical sample consisted of 5,611 participants who had answered either wave three or wave four by February 24, 2020. For simplicity, we refer to wave three as the ‘baseline’ and wave four as the ‘follow-up’.

Participants' mean age was 25.47 years old (SD = 1.26) at baseline and 28.25 (SD = 1.26) at follow-up, indicating a mean period of 33.5 months (SD = 3.82) between assessments. The sample was composed of 57.5% (*n* = 3,227) participants from the French-speaking part of Switzerland and 42.5% (*n* = 2,384) from the German-speaking part.

### Measurements

#### BAs, SUDs and MHPs

All the scales are described in Table 1. Validated German or French versions were available for four scales (gaming, internet, smartphone, CUD); for all others, scales were translated using the forward-backward method by bilingual research team members. Further details and item wordings used can be found in Supplementary Material S1.

**Table 1.** Psychometric scales used to assess the symptom severity of behavioral addictions, substance use disorders, and other mental health problems

	Description and instrument used	Time frame	Indicators	α_bl_	α_fu_
**Behavioral addictions (BA)**					
Gambling	Symptoms of gambling disorder, corresponding to DSM-5 criteria (APA, 2013), assessed with the Pathological Gambling Diagnostic Form (Office of Alcoholism and Substance Abuse Services, 2011). Symptoms include preoccupation, increasing bets, failed attempts to quit, and continued gambling despite negative consequences.	12 M	9 (y/n)	0.843	0.822
Gaming	Symptoms of problematic gaming as assessed with the 7-item Game Addiction Scale (Lemmens, Valkenburg, & Peter, 2009), developed based on DSM-IV criteria for pathological gambling (APA, 1994) and prior research (Griffiths, 2005; Griffiths & Davies, 2005). Symptoms include salience, tolerance, mood modification, relapse, withdrawal, conflict with others and problems.	6 M	7 (L_5_)	0.852	0.838
Internet	Symptoms of problematic internet use as assessed with the Compulsive Internet Use Scale (Meerkerk, Van Den Eijnden, Vermulst, & Garretsen, 2009), inspired by DSM-IV criteria for pathological gambling (APA, 1994) and behavioral addiction criteria (Griffiths, 2005), capturing compulsive or excessive use, difficulty stopping, withdrawal, and interference with daily life.	–	14 (L_5_)	0.922	0.922
Pornography	Symptoms of problematic internet pornography use as assessed with the Online Sexual Compulsivity subscale of the Internet Sex Screening Test (ISST; Delmonico & Miller, 2003), assessing excessive or uncontrollable online sexual activity associated with distress or negative consequences in personal, social, or occupational domains.	12 M	6 (y/n)	0.634	0.659
Smartphone	Symptoms of problematic smartphone use as assessed with the Short Version of the Smartphone Addiction Scale (SAS-SV; Haug et al., 2015; Kwon, Kim, Cho, & Yang, 2013). The SAS-SV is inspired by (Griffiths, 2005) components model and covers daily-life disturbance, positive anticipation, withdrawal, cyberspace-oriented relationships, overuse, and tolerance.	–	10 (L_6_)	0.878	0.871
Work	Symptoms of work addiction as assessed with the Bergen Work Addiction Scale (BWAS; Andreassen et al., 2012), reflecting the six components of addiction (Griffiths, 2005)—salience, mood modification, tolerance, withdrawal, conflict, and relapse—and additionally including negative consequences.	12 M	7 (L_5_)	0.783	0.805
**Substance use disorders (SUD)**					
Alcohol use disorder (AUD)	Symptoms of alcohol use disorder assessed using the DSM-5 criteria (APA, 2013), with items from Knight et al. (2002) and Grant et al. (2003)	12 M	11 (y/n)	0.699	0.713
Cannabis use disorder (CUD)	Symptoms of cannabis use disorder assessed with the Revised Cannabis Use Disorder Identification Test (CUDIT-R; Annaheim, Scotto, & Gmel, 2010), a screening tool based on the AUDIT (Saunders, Aasland, Babor, De la Fuente, & Grant, 1993) and adapted for cannabis use (Adamson & Sellman, 2003). The CUDIT-R captures cannabis consumption, patterns of use, and cannabis-related problems (Annaheim et al., 2010).	12 M	10 (v)	0.898	0.898
**Mental health problems (MHP)**					
Major depressive disorder (MDD)	Symptoms of major depressive disorder assessed with the Major Depression Inventory (WHO-MDI; Bech, Rasmussen, Olsen, Noerholm, & Abildgaard, 2001; Bech, Timmerby, Martiny, Lunde, & Søndergaard, 2015; Olsen, Jensen, Noerholm, Martiny, & Bech, 2003), covering the full spectrum of symptoms defined in DSM-IV (APA, 1994) and ICD-10 (World Health Organization, 1992).	2 W	10 (L_6_)	0.898	0.908
Attention deficit hyperactivity disorder (ADHD)	Symptoms of attention-deficit/hyperactivity disorder (ADHD) assessed with the Adult ADHD Self-Report Scale (ASRS-v1.1; Kessler et al., 2005), capturing DSM-IV Criterion A symptoms of adult ADHD (APA, 1994).	12 M	6 (L_5_)	0.778	0.743
Borderline pers. disorder (BPD)	Symptoms of borderline personality disorder assessed with the McLean Screening Instrument for Borderline Personality Disorder (MSI-BPD; Melartin, Häkkinen, Koivisto, Suominen, & Isometsä, 2009; Zanarini et al., 2003), based on DSM-IV diagnostic criteria (APA, 1994).	LT	10 (t/f)	0.792	0.795
Social anxiety disorder (SAD)	Symptoms of social anxiety disorder assessed with the Clinically Useful Social Anxiety Disorder Outcome Scale (CUSADOS; Dalrymple et al., 2013), developed based on diagnostic interviews including the Structured Clinical Interview for DSM-IV (SCID; First, Spitzer, Gibbon Miriam, & Janet, 1997) and the Psychiatric Diagnostic Screening Questionnaire (PDSQ; Zimmerman & Mattia, 2001).	1 W	12 (L_5_)	0.929	0.929

*Note*: BAs, SUDs, and MHPs were modeled as continuous latent variables indicating symptom severity. Further details on psychometric scales can be found in Supplementary Material S1: Psychometric scales.

Time frame = denoted as reference period in number of months (M), weeks (W), or not explicitly specified (−); indicators = denotes the count of indicators for the latent variables and their response format, where y/n = yes/no; *L*_x_ = Likert-Scale with x points; var = various response options depending on item; α_bl/fu_ = Cronbach's α at baseline (bl) and follow up (fu).

#### Covariates

Participants' age, linguistic region (coded 0 for French-speaking and 1 for German-speaking), and time between baseline and follow-up assessments (in months) were used for adjustments.

### Statistical analyses

BAs (gambling, gaming, internet, pornography, smartphone, work), SUDs (AUD, CUD) and MHPs (MDD, ADHD, BPD, SAD) were conceptualized as continuous latent variables, which were used in all the models to indicate symptom severity. Pearson's correlations were used to describe cross-sectional associations at baseline and at follow-up, as well as stability over time.

Figure 1 illustrates the latent change score (LCS) models with cross-domain coupling (Kievit et al., 2018) that were used to examine pairwise, bidirectional associations between each of the six BAs and other BAs, SUDs, and MHPs. To account for potential confounding, latent change scores were adjusted using observed variables for age at baseline (age_bl_) and linguistic region, as both have been linked to variations in BAs, SUDs, and MHPs (Gmel et al., 2015; Studer, Mohler-Kuo, et al., 2013; Wicki, Marmet, Studer, Epaulard, & Gmel, 2021). In addition, models were adjusted for the time interval between baseline and follow-up (time_bl-fu_). The δs represent cross-lagged coefficients quantifying the degree to which one variable's initial level (e.g., gambling disorder) was associated with a change in another variable (e.g., Δ MDD). The γs are the paths from the baseline measurement to the corresponding change (e.g., gambling disorder at baseline to Δ gambling disorder). These γ-paths adjust for regression to the mean, where individuals with higher baseline measurements in each domain typically exhibit lower measurements at subsequent follow-ups, and vice versa (Gmel, Wicki, Rehm, & Heeb, 2008), which results in a negative association between initial status and the change (Chiolero, Paradis, Rich, & Hanley, 2013). The ζ-paths adjust for covariates (age, linguistic region, time). Finally, the correlation τ expresses mutual changes between two variables (e.g., Δ gambling disorder and Δ MDD). To ensure meaningful interpretations of changes in the LCS models, measurements must not vary over time (McArdle, 2009; McArdle & Prindle, 2008). To achieve strict invariance, factor loadings, intercepts, residual variances, and latent means were constrained to be equal across both measurement points (Xu et al., 2017).

The full information maximum likelihood approach in Mplus software (Muthén & Muthén, 2017) was used to account for missing values. Indices used to indicate a good fit were the root mean squared error of approximation (RMSEA) of ≤ 0.06, the comparative fit index (CFI) and the Tucker–Lewis index (TLI) of ≥ 0.95. However, an RMSEA ≤ 0.08 and a CFI and TLI ≥ 0.90 are also generally acceptable (Brown, 1993; Hu & Bentler, 1999; Kline, 2023). Standardized τ values can be interpreted as effect sizes, where 0.1, 0.3, and 0.5 correspond to small, medium, and large effects, respectively (Cohen, 1988). For the standardized cross-lagged coefficients (δ-paths), benchmarks of 0.03, 0.07, and 0.12 were used to denote small, medium, and large effect sizes, respectively (Orth et al., 2024).

As a sensitivity analysis to the cross-domain coupling model, we used the better-known standard cross-lagged panel models (see Supplementary Fig. S4.1) to estimate cross-lagged paths (δs) and correlations between the residuals (τ).

### Ethics

The Human Research Ethics Committee of the Canton of Vaud approved the C-SURF study (Protocol No. 15/07). All participants were informed about the study and provided written informed consent. Participants were allowed to end their participation in the study at any time.

## Results

### Sample description

The descriptive characteristics of BAs, SUDs and MHPs are presented in Table 2. For easier referencing, we will now use simplified abbreviations such as ‘internet’ to denote the latent variable of the severity of problematic internet use symptoms. The stability of BA symptom severity (baseline-follow-up correlations) varied across BAs, ranging from 0.588 (work) to 0.738 (pornography), with similar coefficients for established (gambling, gaming) and proposed BAs as assessed with their respective validated self-report instruments. Cross-sectionally significant positive correlations were observed for all BAs, SUDs and MHPs examined at both time points, with the notable exceptions of gambling and ADHD at baseline, work and CUD at baseline, and smartphone use and CUD at follow-up. The percentage of participants who screened positive for BAs, SUDs, and MHPs at baseline and follow-up, according to the respective instrument cut-offs, are provided in Supplementary Table S2.1. All the fit indices for the LCS models were good or at least acceptable (see Supplementary Table S3.1).

**Table 2.** Descriptive characteristics of behavioral addictions, substance use disorders and mental health problems: percentage of sample screened positively, stability of symptom severity between baseline and follow-up, and cross-sectional correlations between the symptoms of mental health problems at baseline and at follow-up

	*n*		Stability	Correlations between latent variables at baseline						Correlations between latent variables at follow-up					
	Baseline	Follow-up		1	2	3	4	5	6	1	2	3	4	5	6
			*r*_*bl-fu*_	*r*	*r*	*r*	*r*	*r*	*r*	*r*	*r*	*r*	*r*	*r*	*r*
**Behavioral addictions**															
1. Gambling	5,505	5,090	0.725***												
2. Gaming	5,511	5,091	0.668***	0.293***						0.317***					
3. Internet	5,512	5,089	0.620***	0.241***	0.501***					0.299***	0.340***				
4. Pornography	5,427	5,090	0.738***	0.234***	0.216***	0.431***				0.263***	0.236***	0.489***			
5. Smartphone	5,513	5,089	0.624***	0.298***	0.234***	0.617***	0.343***			0.361***	0.207***	0.670***	0.360***		
6. Work	5,434	5,052	0.588***	0.189***	0.031^t^	0.189***	0.211***	0.199***		0.203***	0.072***	0.228***	0.218***	0.195***	
**Substance use disorders**															
7. AUD	5,507	5,087	0.704***	0.364***	0.141***	0.277***	0.305***	0.267***	0.148***	0.333***	0.152***	0.277***	0.312***	0.271***	0.153***
8. CUD	5,502	5,090	0.869***	0.145**	0.217***	0.174***	0.205***	0.073**	0.031	0.157*	0.233***	0.101***	0.168***	0.035	0.051*
**Mental health problems**															
9. MDD	5,435	5,054	0.579***	0.305***	0.327***	0.412***	0.325***	0.284***	0.473***	0.341***	0.354***	0.383***	0.330***	0.272***	0.499***
10. ADHD	5,509	5,088	0.563***	0.077^t^	0.286***	0.490***	0.313***	0.357***	0.235***	0.165**	0.335***	0.468***	0.347***	0.367***	0.283***
11. BPD	5,426	5,054	0.695***	0.328***	0.332***	0.371***	0.391***	0.266***	0.337***	0.411***	0.351***	0.337***	0.394***	0.253***	0.361***
12. SAD	5,425	5,053	0.578***	0.296***	0.305***	0.397***	0.289***	0.346***	0.300***	0.358***	0.276***	0.378***	0.331***	0.328***	0.353***

*Note*: BAs, SUDs, and MHPs were modeled as continuous latent variables indicating symptom severity. *n* = participants who completed the respective scale; for correlations, Full Information Maximum Likelihood (FIML) was used, allowing for the inclusion of participants with missing values under the ‘missing at random’ assumption (*n* = 5,611); gambling = gambling disorder; gaming = problematic gaming; internet = problematic internet use; pornography = problematic internet pornography use; smartphone = problematic smartphone use; work = work addiction; ADHD = attention-deficit/hyperactivity disorder. **Bold** font indicates significant coefficients (*p* < 0.050); ***p < 0.001, **p < 0.010, *p < 0.050, ^t^*p* < 0.100.

### Correlated changes (τ) between BAs, and between BAs, SUDs, and MHPs

Correlated changes between BAs, and between BAs and MHPs, were all significant and positive (Table 3); for instance, changes in gambling symptom severity between baseline and follow-up were positively associated with changes in MDD symptom severity over the same period (*r* = 0.247, *p* < 0.001) with a small effect size (S). For SUDs, significant positively correlated changes were found between AUD and all the BAs (except gambling), and between CUD and gaming and work. The largest effect sizes were found among BAs: smartphone and internet use (large, > 0.5), and pornography and internet or gambling (both medium, > 0.3). Between BAs and other MHPs, some correlated changes reached medium effect sizes: MDD and work, and BPD and gambling (both medium, > 0.3). For BAs and SUDs, correlated changes were small or did not reach the threshold for a small effect size.

**Table 3.** Correlated change (τ) between the symptom severity of behavioral addictions and other mental health problems

	Behavioral Addictions (BAs)																							
	Gambling				Gaming				Internet				Pornography				Smartphone				Work			
	*r*	95%CI	*p*		*r*	95%CI	*p*		*r*	95%CI	*p*		*r*	95%CI	*p*		*r*	95%CI	*p*		*r*	95%CI	*p*	
**Behavioral addictions**																								
Gaming	**0.168**	[0.004, 0.332]	0.044	^S^																				
Internet	**0.209**	[0.061, 0.358]	0.006	^S^	**0.235**	[0.185, 0.284]	<0.001	^S^																
Pornography	**0.308**	[0.007, 0.609]	0.045	^M^	**0.134**	[0.062, 0.206]	<0.001	^S^	**0.323**	[0.268, 0.377]	<0.001	^M^												
Smartphone	**0.281**	[0.135, 0.427]	<0.001	^S^	**0.190**	[0.141, 0.240]	<0.001	^S^	**0.572**	[0.544, 0.599]	<0.001	^L^	**0.287**	[0.229, 0.344]	<0.001	^S^								
Work	**0.148**	[0.021, 0.276]	0.023	^S^	**0.052**	[0.008, 0.096]	0.021		**0.134**	[0.097, 0.171]	<0.001	^S^	**0.156**	[0.096, 0.216]	<0.001	^S^	**0.119**	[0.080, 0.157]	<0.001	^S^				
**Substance use disorders**																								
AUD	0.169	[−0.059, 0.396]	0.147	^S^	**0.146**	[0.072, 0.221]	<0.001	^S^	**0.182**	[0.120, 0.243]	<0.001	^S^	**0.253**	[0.159, 0.347]	<0.001	^S^	**0.211**	[0.151, 0.270]	<0.001	^S^	**0.127**	[0.060, 0.193]	<0.001	^S^
CUD	0.064	[−0.195, 0.322]	0.629		**0.153**	[0.061, 0.244]	0.001	^S^	0.019	[−0.061, 0.099]	0.637		0.121	[−0.012, 0.254]	0.076	^S^	0.007	[−0.077, 0.092]	0.865		**0.094**	[0.011, 0.178]	0.027	
**Mental health problems**																								
MDD	**0.247**	[0.124, 0.371]	<0.001	^S^	**0.237**	[0.195, 0.280]	<0.001	^S^	**0.228**	[0.192, 0.264]	<0.001	^S^	**0.181**	[0.117, 0.246]	<0.001	^S^	**0.178**	[0.140, 0.217]	<0.001	^S^	**0.393**	[0.360, 0.425]	<0.001	^M^
ADHD	**0.253**	[0.100, 0.405]	0.001	^S^	**0.212**	[0.163, 0.261]	<0.001	^S^	**0.277**	[0.241, 0.313]	<0.001	^S^	**0.215**	[0.153, 0.276]	<0.001	^S^	**0.248**	[0.209, 0.286]	<0.001	^S^	**0.225**	[0.189, 0.261]	<0.001	^S^
BPD	**0.348**	[0.186, 0.510]	<0.001	^M^	**0.218**	[0.161, 0.275]	<0.001	^S^	**0.212**	[0.164, 0.261]	<0.001	^S^	**0.265**	[0.189, 0.342]	<0.001	^S^	**0.210**	[0.161, 0.260]	<0.001	^S^	**0.264**	[0.217, 0.311]	<0.001	^S^
SAD	**0.266**	[0.122, 0.409]	<0.001	^S^	**0.211**	[0.166, 0.255]	<0.001	^S^	**0.239**	[0.203, 0.274]	<0.001	^S^	**0.241**	[0.181, 0.301]	<0.001	^S^	**0.231**	[0.193, 0.270]	<0.001	^S^	**0.248**	[0.212, 0.284]	<0.001	^S^

*Note*: BAs, SUDs, and MHPs were modeled as continuous latent variables indicating symptom severity. Gambling = gambling disorder; gaming = problematic gaming; internet = problematic internet use; pornography = problematic internet pornography use; smartphone = problematic smartphone use; work = work addiction; MHPs = mental health problems; ADHD = attention-deficit/hyperactivity disorder; *r* = Pearson correlation coefficient; CI = confidence interval.

Full Information Maximum Likelihood (FIML) was used, allowing for the inclusion of participants with missing values under the ‘missing at random’ assumption (*n* = 5,611). **Bold** font indicates significant coefficients (*p* < 0.050). ^S/M/L^ = small/medium/large effect size according to Cohen (1988).

### Directional changes (δ) between BAs, and between BAs, SUDs, and MHPs

Directional changes between BAs and MHPs were largely positive and bidirectional, significant for 15 of 30 tested paths (Table 4). For instance, the more severe pornography symptoms were at baseline, the greater the increase (or attenuation of decrease) in internet symptom severity between baseline and follow-up, and vice versa (*δ*_1_ = 0.155, *p* < 0.001; *δ*_2_ = 0.140, *p* < 0.001), both with large effect sizes (L). Often, these directional changes were mutually significant (e.g., pornography and internet); in some instances, only one of the two directional changes was significant, but overlapping 95% confidence intervals (95%CIs) indicated no significant difference (e.g., for gambling and gaming). Negative directional changes were only found for work and CUD, and for gaming and internet. For instance, the more severe work symptoms were at baseline, the smaller the increase in CUD symptoms between baseline and follow-up (*δ*_2_ = 0.073, *p* = 0.040).

**Table 4.** Directional change (δ_1_ and δ_2_) between the symptom severity of behavioral addictions and other mental health problems, standardized path coefficients

	Pathway	Behavioral Addictions (BAs)																							
		Gambling				Gaming				Internet				Pornography				Smartphone				Work			
		*β*	95%CI	*p*		*β*	95%CI	*p*		*β*	95%CI	*p*		*β*	95%CI	*p*		*β*	95%CI	*p*		*β*	95%CI	*p*	
**Behavioral addictions**																									
Gaming	δ_1 Gaming bl →_ _ΔBA_	**0.158**	[0.029, 0.288]	0.017	^L^																				
	δ_2 BA bl →_ _ΔGaming_	0.014	[−0.109, 0.137]	0.821																					
Internet	δ_1 Internet bl →_ _ΔBA_	**0.129**	[0.013, 0.245]	0.029	^L^	**0.070**	[0.026, 0.114]	0.002	^M^																
	δ_2 BA bl →_ _ΔInternet_	0.029	[−0.067, 0.125]	0.553		**−0.088**	[−0.132, −0.044]	<0.001	^M^																
Pornography	δ_1 Pornography bl →_ _ΔBA_	0.059	[−0.082, 0.200]	0.412	^S^	**0.063**	[0.009, 0.118]	0.023	^S^	**0.155**	[0.107, 0.203]	<0.001	^L^												
	δ_2 BA bl →_ _ΔPornography_	−0.079	[−0.259, 0.101]	0.389	^M^	0.054	[−0.006, 0.115]	0.080	^S^	**0.140**	[0.081, 0.200]	<0.001	^L^												
Smartphone	δ_1 Smartphone bl →_ _ΔBA_	**0.156**	[0.035, 0.277]	0.011	^L^	−0.022	[−0.065, 0.020]	0.306		**0.152**	[0.110, 0.194]	<0.001	^L^	0.055	[−0.005, 0.115]	0.073	^S^								
	δ_2 BA bl →_ _ΔSmartphone_	0.025	[−0.069, 0.119]	0.603		0.025	[−0.012, 0.061]	0.190		**0.147**	[0.108, 0.186]	<0.001	^L^	**0.063**	[0.017, 0.109]	0.007	^S^								
Work	δ_1 Work bl →_ _ΔBA_	−0.027	[−0.143, 0.088]	0.642		0.003	[−0.036, 0.042]	0.881		**0.109**	[0.076, 0.141]	<0.001	^M^	0.052	[−0.006, 0.109]	0.077	^S^	**0.046**	[0.012, 0.080]	0.008	^S^				
	δ_2 BA bl →_ _ΔWork_	0.067	[−0.014, 0.149]	0.107	^S^	0.024	[−0.012, 0.061]	0.184		**0.045**	[0.012, 0.078]	0.008	^S^	0.044	[−0.002, 0.089]	0.061	^S^	**0.062**	[0.028, 0.096]	<0.001	^S^				
**Substance use disorders**																									
AUD	δ_1 AUD bl →_ _ΔBA_	0.157	[−0.025, 0.339]	0.091	^L^	0.016	[−0.036, 0.067]	0.547		**0.061**	[0.013, 0.108]	0.013	^S^	0.021	[−0.074, 0.117]	0.659		**0.060**	[0.014, 0.105]	0.010	^S^	0.025	[−0.020, 0.069]	0.275	
	δ_2 BA bl →_ _Δ AUD_	−0.044	[−0.208, 0.119]	0.595	^S^	−0.018	[−0.077, 0.041]	0.547		0.029	[−0.027, 0.085]	0.304		0.026	[−0.048, 0.099]	0.493		−0.002	[−0.057, 0.053]	0.947		−0.004	[−0.059, 0.051]	0.888	
CUD	δ_1 CUD bl →_ _ΔBA_	0.096	[−0.072, 0.264]	0.262	^M^	**0.085**	[0.028, 0.141]	0.003	^M^	0.011	[−0.037, 0.058]	0.660		0.040	[−0.038, 0.118]	0.311	^S^	0.002	[−0.044, 0.048]	0.945		0.024	[−0.017, 0.065]	0.256	
	δ_2 BA bl →_ _ΔCUD_	−0.061	[−0.261, 0.140]	0.552	^S^	−0.039	[−0.119, 0.040]	0.333	^S^	−0.029	[−0.097, 0.039]	0.404		−0.085	[−0.186, 0.017]	0.102	^M^	−0.026	[−0.093, 0.042]	0.454		**−0.073**	[−0.143, −0.003]	0.040	^M^
**Mental health problems**																									
MDD	δ_1 MDD bl →_ _ΔBA_	0.034	[−0.087, 0.154]	0.584	^S^	**0.074**	[0.032, 0.115]	<0.001	^M^	**0.092**	[0.056, 0.127]	<0.001	^M^	**0.087**	[0.029, 0.145]	0.003	^M^	**0.078**	[0.044, 0.111]	<0.001	^M^	**0.087**	[0.050, 0.124]	<0.001	^M^
	δ_2 BA bl →_ _Δ MDD_	**0.096**	[0.009, 0.183]	0.030	^M^	**0.082**	[0.044, 0.119]	<0.001	^M^	**0.129**	[0.094, 0.163]	<0.001	^L^	**0.115**	[0.068, 0.162]	<0.001	^M^	**0.061**	[0.028, 0.095]	<0.001	^S^	**0.109**	[0.072, 0.146]	<0.001	^M^
ADHD	δ_1 ADHD bl →_ _ΔBA_	−0.051	[−0.181, 0.079]	0.442	^S^	**0.077**	[0.034, 0.121]	<0.001	^M^	**0.100**	[0.062, 0.139]	<0.001	^M^	**0.103**	[0.038, 0.167]	0.002	^M^	**0.074**	[0.038, 0.110]	<0.001	^M^	−0.015	[−0.050, 0.019]	0.383	
	δ_2 BA bl →_ _ΔADHD_	0.002	[−0.078, 0.082]	0.967		**0.088**	[0.053, 0.124]	<0.001	^M^	**0.183**	[0.150, 0.215]	<0.001	^L^	**0.097**	[0.053, 0.141]	<0.001	^M^	**0.109**	[0.076, 0.142]	<0.001	^M^	**0.097**	[0.066, 0.129]	<0.001	^M^
BPD	δ_1 BPD bl →_ _ΔBA_	0.060	[−0.072, 0.193]	0.370	^S^	**0.087**	[0.039, 0.135]	<0.001	^M^	**0.076**	[0.034, 0.118]	<0.001	^M^	0.047	[−0.025, 0.119]	0.198	^S^	**0.047**	[0.009, 0.086]	0.017	^S^	**0.090**	[0.049, 0.130]	<0.001	^M^
	δ_2 BA bl →_ _Δ BPD_	0.059	[−0.078, 0.197]	0.398	^S^	**0.057**	[0.007, 0.107]	0.025	^S^	0.033	[−0.013, 0.080]	0.159	^S^	**0.088**	[0.023, 0.153]	0.008	^M^	−0.013	[−0.059, 0.034]	0.586		0.037	[−0.010, 0.084]	0.124	^S^
SAD	δ_1 SAD bl →_ _ΔBA_	0.060	[−0.060, 0.181]	0.326	^S^	0.001	[−0.042, 0.045]	0.953		**0.077**	[0.042, 0.112]	<0.001	^M^	**0.059**	[0.003, 0.115]	0.039	^S^	**0.084**	[0.049, 0.118]	<0.001	^M^	**0.081**	[0.047, 0.115]	<0.001	^M^
	δ_2 BA bl →_ _ΔSAD_	**0.098**	[0.017, 0.179]	0.018	^M^	0.035	[−0.002, 0.073]	0.062	^S^	**0.106**	[0.072, 0.140]	<0.001	^M^	**0.086**	[0.044, 0.129]	<0.001	^M^	**0.047**	[0.013, 0.082]	0.007	^S^	**0.097**	[0.063, 0.131]	<0.001	^M^

*Note*: BAs, SUDs, and MHPs were modeled as continuous latent variables indicating symptom severity. Gambling = gambling disorder; gaming = problematic gaming; internet = problematic internet use; pornography = problematic internet pornography use; smartphone = problematic smartphone use; work = work addiction; AUD = alcohol use disorder; CUD = cannabis use disorder; MDD = major depressive disorder; ADHD = attention-deficit/hyperactivity disorder; BPD = borderline personality disorder; SAD = social anxiety disorder; Δ = latent change; δ_1_ = pathway between BA, SUD, or MHP at baseline and latent change in BA between baseline and follow-up; δ_2_ = pathway between behavioral addiction BA at baseline and latent change in BA, SUD, or MHP between baseline and follow-up. *β* = standardized path coefficient; CI = confidence interval.

Full Information Maximum Likelihood (FIML) was used, allowing for the inclusion of participants with missing values under the ‘missing at random’ assumption (*n* = 5,611). **Bold** font indicates significant coefficients (*p* < 0.050). ^S/M/L^ = small/medium/large effect size according to Orth et al. (2024).

Between BAs and MHPs, directional changes were particularly common, and all significant (35 out of 48 tested) associations were positive. Most BA–MHP pairs showed consistent effects in both directions — either both paths were significant (e.g., gaming and MDD) or their 95%CIs overlapped (e.g., gambling and MDD). The only unidirectional effect was observed for work and ADHD, with non-overlapping 95%CIs. However, no directional change was found between gambling and ADHD, gambling and BPD, and gaming and SAD.

Between BAs directional changes were comparatively less common, with 15 of the 30 tested pathways reaching significance. Internet use showed directional associations with each of the other BAs. Additional significant directional changes were found for smartphone—with gambling, pornography, and work—and for gaming with gambling and pornography, but not for work with gambling, gaming, or pornography.

Between BAs and SUDs directional changes were least common, with only 4 of the 20 tested pathways reaching significance. In three cases, higher baseline SUD symptoms predicted an increase in BA symptom severity over time (AUD with internet, AUD with smartphone, and CUD with gaming). One additional significant path indicated that more severe work symptoms at baseline predicted a decrease in CUD symptom severity from baseline to follow-up.

### Sensitivity analysis

Findings from the sensitivity analysis, made using a cross-lagged panel model, were largely comparable with the findings from the main analysis using LCS models (see Supplementary Fig. S4.1 and Tables S4.1–S4.3).

## Discussion

The aim of this study was to examine correlated and directional changes in symptom severity between BAs, and between BAs, SUDs, and MHPs, over an average period of almost three years in a non-self-selecting cohort of young Swiss men. By applying the same longitudinal modeling approach across a range of BAs, SUDs, and MHPs within a single sample, this study offers a rare opportunity to directly compare temporal patterns across domains.

### Findings for aim 1: correlated change

In line with previous studies (Colder Carras, Shi, Hard, & Saldanha, 2020; Kim et al., 2024; Ostinelli et al., 2021; Wang, Yin, Wang, King, & Rost, 2024), we observed that changes in the symptom severity of all six BAs studied were positively correlated with changes in the symptom severity of other BAs, AUD, and MHPs. For CUD, correlated changes were less consistent and often failed to reach significance. While the latter may appear to contradict other studies showing such associations (Dash et al., 2019; Forsyth & Malone, 2016; Van Rooij et al., 2014), this could possibly be due to limited symptom variations in CUD, as reflected in its high temporal stability (*r* = 0.869).

### Findings for aim 2: directional change

We also examined directional change, testing the baseline symptom severity of BAs predicted subsequent changes in the symptom severity of other BAs, SUDs, and MHPs over time, and vice versa. Overall, reciprocal changes were most consistent between BAs and MHPs, followed by BAs using similar media (e.g., internet, pornography, smartphones), and least consistent between BAs and SUDs.

Between BAs and MHPs, reciprocal directional changes were consistently observed, as proposed by Brand et al.’s I-PACE model (2025)—i.e., between variables linked through positive feedback loops. Contrary to earlier studies (Brunault, Mathieu, Faussat, Barrault, & Varescon, 2020; Kaleda, Krylova, Kuleshov, & Beburishvili, 2021), no directional change was found for gambling and ADHD or BPD, and for gaming and SAD. This may be due to mixed patterns of behavioral evolution—for some individuals, the social anxiety initially associated with gaming may improve following in-game social interactions (Gioia, Colella, & Boursier, 2022)—and the relatively low percentage of people with both conditions.

Finally, our findings concerning rarely significant directional changes between BAs and SUDs (Gomez et al., 2022) is consistent with the I-PACE model (Brand et al., 2025), which allows for shared risk factors (Lieb, 2014), without implying mutual reinforcement between these domains.

### Implications for theory and interventions

Our use of directional changes as indicators of potential causal relationships (Klimstra, Bleidorn, Asendorpf, van Aken, & Denissen, 2013; Lieb, 2014; Littlefield et al., 2009, 2012) contributes to understanding the temporal dynamics linking BAs, SUDs, and MHPs, advancing beyond cross-sectional research. The observed directional changes, particularly between BAs and MHPs, support the I-PACE model's assumption of reciprocal feedback loops between person-related factors (e.g., MDD, ADHD, SAD) and the development or maintenance of BAs (Brand et al., 2025). However, further research is needed to examine affective and cognitive responses as well as executive control (additional core components of the I-PACE model) to further capture the mechanisms underlying BAs. Nonetheless, the consistency of patterns across both established (e.g., gambling) and emerging (e.g., internet, pornography, smartphone use) BAs suggests shared mechanisms and may have implications for the classification and clinical recognition of emerging BAs.

Reciprocal between-BA changes were most consistently observed in associations involving problematic internet or smartphone use, two partially overlapping vectors of specific addictive behaviors (Montag et al., 2021). This underscores the phenotypical closeness of internet- and smartphone-based BAs and suggest that the technologies themselves can serve not only as platforms, but also as amplifiers of behaviors such as gambling, gaming, or pornography use (Khazaal et al., 2015; Starcevic & Billieux, 2017). The constant availability, immediacy, and interactive reinforcement built into these technologies may partly explain the directional changes observed between behaviors on these platforms— for example, porn games and loot boxes in games (Spicer et al., 2022; Zendle, Meyer, Cairns, Waters, & Ballou, 2020). These patterns point to screen-based technologies as a shared context that shapes affective and cognitive responses and facilitates transitions between BAs, aligning with mechanisms described in the I-PACE model (Brand et al., 2025). More broadly, they highlight the importance of considering the medium as a component of the addictive process.

In contrast to other BAs, work addiction showed a distinct pattern of associations, suggesting it may represent a separate subtype (cf. Griffiths et al., 2018). Unlike other BAs, it appears more achievement-oriented and is linked to traits like perfectionism and conscientiousness, which tend to show negative associations with other BAs (Andreassen et al., 2013; Atroszko, 2022; Clark et al., 2016). High work involvement is often socially accepted and rewarded, whereas high involvement in behaviors such as gambling, gaming, or pornography use tends to be stigmatized (Casale et al., 2023; Grubbs, Exline, Pargament, Hook, & Carlisle, 2015; Peter, Li, Pfund, Whelan, & Meyers, 2019). Incompatibilities between work addiction and other BAs or SUDs (e.g., due to time constraints or the avoidance of hangovers), combined with its social desirability, may reflect a “mixed blessing” dynamic (Brown, 1993), where overinvolvement in work brings both strain and benefits. As no established theoretical model explains the biological underpinnings of work addiction (Atroszko, 2022), the I-PACE model (Brand et al., 2025) may offer a starting point for conceptualizing its psychological mechanisms, though adaptations are needed to capture its social desirability, identity-related motives, and achievement-based reinforcement.

Given the reciprocal directional changes between BAs and MHPs, our findings suggest not only the presence of self-reinforcing circles of disfunction but also the potential for virtuous loops—where improvement in one domain facilitates gains in another. Interventions targeting either BAs or MHPs may yield cross-domain effects, and structured programs could leverage these dynamics by addressing both simultaneously, underscoring the value of integrated approaches in disrupting maladaptive cycles and supporting sustained recovery. The observed interrelations support transdiagnostic perspectives on BAs and underscore the need for prevention and intervention programs that address the addictive behavior and co-occurring MHPs. Integrated treatment plans that consider these aspects alongside psychosocial factors may prove particularly effective in managing such complex cases (Dom & Moggi, 2014; Morisano et al., 2014; van Wamel et al., 2014). Moreover, prevention and informational efforts in high-risk environments (e.g., casinos, online gambling platforms) could move beyond targeting addictive behaviors alone to also consider potential co-occurring mental health problems and related psychosocial factors.

### Limitations

This study had some limitations that should be considered when interpreting the results. Firstly, generalizations should be made with caution, and analyses should be replicated and extended across different sociodemographic categories (e.g., women, other age groups), ethnocultural backgrounds (e.g., non-Swiss), and populations (e.g., treatment-seeking individuals; Kowalewska, Bőthe, & Kraus, 2024; Pontes et al., 2022). Further research is particularly needed on gender, which is known to affect both the prevalence of BAs (Laskowski, Hildebrandt, & Muschalla, 2024; Merkouris et al., 2016; Stevens, Dorstyn, Delfabbro, & King, 2021; Su, Han, Yu, Wu, & Potenza, 2020) and, in some cases, the psychological processes described in the I-PACE model (Brand et al., 2025); while some studies report largely comparable processes across genders (Marino et al., 2023; Pekal, Laier, Snagowski, Stark, & Brand, 2018), others highlight gender-specific patterns (Dong, Wang, Du, & Potenza, 2018; Jhone, Song, Lee, Yoon, & Bhang, 2021; Su, Király, Demetrovics, & Potenza, 2019). Secondly, self-reporting screening instruments, while useful for large-scale data collection, can introduce biases like social desirability and recall inaccuracies, potentially misrepresenting clinical realities. These biases may lead to underreporting or misreporting. Future research should validate self-reported data against clinical diagnoses or use ecological momentary assessment to enhance data accuracy. Thirdly, some instruments had no validated German or French versions and were translated by bilingual team members using a forward–backward procedure. Furthermore, to ensure comparability across waves, some instruments aligned with earlier versions of diagnostic criteria, which may limit generalizability to current diagnostic frameworks. Finally, our analysis assumed normality and linearity when conceptualizing changes, without testing for potential subgroup differences or non-linear effects across varying symptom severities. Although a reduction in the symptom severity of a BA was typically associated with a reduction in the symptom severity of other MHPs, this trend may not apply universally to all individuals or subgroups; some may exhibit distinct patterns of change (Kim et al., 2021; Sinclair et al., 2021).

## Conclusion

This study provides strong evidence that BAs and MHPs mutually reinforce each other over time. The findings support the applicability of theoretical models such as I-PACE (Brand et al., 2025) by emphasizing the dynamic interplay between person-related predisposing factors (e.g., MDD, ADHD), situational triggers, and addictive behaviors over time, to both established BAs (e.g., gambling, gaming) and proposed ones (e.g., problematic internet, pornography, or smartphone use). Importantly, while mutually reinforcing cycles between BAs and MHPs can maintain dysfunction, they also offer potential for virtuous loops, where improvements in one domain may catalyze positive changes in another. Integrated intervention plans that address both addictive behaviors and co-occurring mental health problems may therefore be particularly effective in breaking maladaptive cycles and supporting sustainable recovery.

## Supplementary data

## Data Availability

The datasets analyzed for this study can be found on Zenodo (https://zenodo.org/records/5469953).
