# Supplementary material for: Behavioral addictions and their reciprocal associations with each other, substance use disorders, and mental health problems: Findings from a longitudinal cohort study of young Swiss men
Source: J Behav Addict. 2025 Sep 15;14(3):1250–66. doi: 10.1556/2006.2025.00078 (PMC12486285; doi:10.1556/2006.2025.00078)
Supplement: Supplementary file 1 [file jba-14-1250-s001.pdf]

**Wicki, M. et al.: Behavioral addictions and their reciprocal associations with each other, substance use disorders, and mental health problems: Findings from a longitudinal cohort study of young Swiss men**

https://doi.org/10.1556/2006.2025.00078

# SUPPLEMENTARY MATERIAL

## Supplementary Material S1: Psychometric scales

This is a short description of the psychometric scales used to assess the symptom severity of behavioral addictions (BAs), substance use disorders (SUDs), and other mental health problems (MHPs) reported in Table 1 of the main text of the paper.

### *S1.1 Gambling*

The study assessed symptoms of gambling disorder in accordance with the DSM-5 criteria for gambling disorder (American Psychiatric Association [APA], 2013) and nine items from the DSM-IV Pathological Gambling Diagnostic Form (APA, 1994; Office of Alcoholism and Substance Abuse Services, 2011) were used. To align the instrument with DSM-5 criteria for gambling disorder (APA, 2013), the item addressing “going beyond what is strictly legal” was removed.

The French and German versions were translated by bilingual members of the research team using the forward-backward translation method.

**English**
IN THE PAST 12 MONTHS…

| | *Tick one box in each row* | **Yes** *1* | **No** *0* |
|---|---|---|---|
| 1. | … have you often found yourself thinking about gambling (e.g. reliving past gambling experiences, planning the next time you will play or thinking of ways to get money to gamble)? | ☐ | ☐ |
| 2. | … have you needed to gamble with more and more money to get the amount of excitement you are looking for? | ☐ | ☐ |
| 3. | … have you become restless or irritable when trying to cut down or stop gambling? | ☐ | ☐ |
| 4. | … have you gambled to escape from problems or when you are feeling depressed, anxious or bad about yourself? | ☐ | ☐ |
| 5. | … after losing money gambling, have you returned another day in order to get even? | ☐ | ☐ |
| 6. | … have you lied to your family or others to hide the extent of your gambling? | ☐ | ☐ |
| 7. | … have you made repeated unsuccessful attempts to control, cut back or stop gambling? | ☐ | ☐ |
| 8. | … have you risked or lost a significant relationship, job, educational or career opportunity because of gambling? | ☐ | ☐ |
| 9. | … have you sought help from others to provide the money to relieve a desperate financial situation caused by gambling? | ☐ | ☐ |

**French**

AU COURS des 12 DERNIERS MOIS…

| | *Cochez une case par ligne.* | Oui<br>*1* | Non<br>*0* |
|---|---|---|---|
| 1. | …vous êtes-vous souvent trouvé à penser aux jeux d'argent (p. ex. se remémorer des expériences de jeu passées, planifier de nouvelles tentatives, ou penser aux moyens d'obtenir de l'argent pour jouer) ? | ☐ | ☐ |
| 2. | …avez-vous eu besoin de jouer de plus en plus d'argent pour obtenir l'état d'excitation que vous recherchez ? | ☐ | ☐ |
| 3. | …êtes-vous devenu agité ou irritable lorsque vous avez essayé de diminuer ou d'arrêter de jouer ? | ☐ | ☐ |
| 4. | …avez-vous joué à des jeux d'argent pour fuir des problèmes ou pour atténuer des sentiments négatifs (p.ex., sentiment d'impuissance, culpabilité, anxiété, dépression) ? | ☐ | ☐ |
| 5. | …après avoir perdu de l'argent au jeu, êtes-vous retourné jouer le lendemain pour recouvrer vos pertes (pour vous « refaire ») ? | ☐ | ☐ |
| 6. | …avez-vous menti plusieurs fois à votre famille ou à d'autres pour dissimuler l'ampleur de vos habitudes de jeu ? | ☐ | ☐ |
| 7. | …avez-vous entrepris des tentatives répétées mais infructueuses pour contrôler, réduire ou arrêter de jouer ? | ☐ | ☐ |
| 8. | …avez-vous mis en danger ou perdu une relation affective importante, un emploi, une perspective de formation ou de carrière à cause du jeu ? | ☐ | ☐ |
| 9. | …avez-vous demandé une aide financière auprès de tiers pour remédier à une situation financière désastreuse due au jeu ? | ☐ | ☐ |

**German**

In den LETZTEN 12 MONATEN ….

| | *Kreuzen Sie in jeder Zeile ein Kästchen an.* | Ja<br>*1* | Nein<br>*0* |
|---|---|---|---|
| 1. | …waren Sie jemals über mehrere Tage häufig oder ständig gedanklich mit dem Spielen beschäftigt (z.B. Erinnerung an Glücksspielerlebnisse, Planung des nächsten Spiels, Nachdenken über Geldbeschaffung für das Spielen)? | ☐ | ☐ |
| 2. | …mussten Sie mit immer höheren Einsätzen spielen, um den gewünschten „Kick" zu erreichen? | ☐ | ☐ |
| 3. | …waren Sie unruhig und gereizt beim Versuch, das Spielen einzuschränken oder aufzugeben? | ☐ | ☐ |
| 4. | …haben Sie gespielt, um Problemen zu entkommen oder um negative Gefühlszustände (z.B. Gefühle von Hilflosigkeit, Schuld, Angst, Depression) zu verringern? | ☐ | ☐ |
| 5. | …haben Sie jemals gezielt am darauffolgenden Tag gespielt, um zuvor verlorenes Geld wieder zurückzugewinnen? | ☐ | ☐ |
| 6. | …haben Sie schon mehrmals Ihren Angehörigen, Freunden oder anderen Personen nicht die Wahrheit gesagt, um das Ausmaß Ihres Glücksspielverhaltens zu verheimlichen? | ☐ | ☐ |
| 7. | …haben Sie wiederholt erfolglose Versuche unternommen, das Spielen zu kontrollieren, einzuschränken oder aufzugeben? | ☐ | ☐ |
| 8. | …haben Sie eine wichtige Beziehung, Ihren Arbeitsplatz, Ausbildungs- oder Aufstiegschancen wegen des Spielens gefährdet oder verloren? | ☐ | ☐ |
| 9. | …haben Sie sich finanzielle Hilfe gesucht, um die durch das Spielen verursachte hoffnungslose finanzielle Situation zu überwinden? | ☐ | ☐ |

### *S1.2 Gaming*

The study assessed symptoms of problematic gaming, as conceptualized by the 7-item Game Addiction Scale (Lemmens et al., 2009). The scale was developed by adapting the DSM-IV criteria for pathological gambling (APA, 1994) to the context of gaming and by drawing on prior research (e.g., Griffiths, 2005; Griffiths & Davies, 2005).

The Game Addiction Scale (Lemmens et al., 2009) refers to both online and offline gaming, similarly to the later definition of "gaming disorder" in the ICD-11 (World Health Organization, 2018), and in contrast to the DSM-5, which introduced "Internet Gaming Disorder" in Section III as a condition warranting further study, with a focus specifically on online gaming (APA, 2013).

The French and German versions were translated by bilingual members of the research team using the forward-backward translation method and validated by Khazaal et al. (2016).

English

IN THE PAST 6 MONTHS, how often…

| | *Tick one box in each row.* | **Never** *1* | **Rarely** *2* | **Some-times** *3* | **Often** *4* | **Very often** *5* |
|---|---|---|---|---|---|---|
| 1. | …have you thought all day long about playing a game? | ☐ | ☐ | ☐ | ☐ | ☐ |
| 2. | …have you played longer than intended? | ☐ | ☐ | ☐ | ☐ | ☐ |
| 3. | …have you played games to forget about real life? | ☐ | ☐ | ☐ | ☐ | ☐ |
| 4. | …have others unsuccessfully tried to make you reduce your time spent on games? | ☐ | ☐ | ☐ | ☐ | ☐ |
| 5. | …have you felt upset when you were unable to play? | ☐ | ☐ | ☐ | ☐ | ☐ |
| 6. | …have you had arguments with others (e.g., family, friends) over your time spent on games? | ☐ | ☐ | ☐ | ☐ | ☐ |
| 7. | …have you neglected important activities (e.g. school, work, sports) to play games? | ☐ | ☐ | ☐ | ☐ | ☐ |

**French**

AU COURS DES 6 DERNIERS MOIS, à quelle fréquence…

| | *Cochez une case par ligne.* | **Jamais** *1* | **Rarement** *2* | **Parfois** *3* | **Souvent** *4* | **Très souvent** *5* |
|---|---|---|---|---|---|---|
| 1. | … avez-vous pensé à jouer durant toute la journée ? | ☐ | ☐ | ☐ | ☐ | ☐ |
| 2. | … avez-vous joué plus longtemps que prévu ? | ☐ | ☐ | ☐ | ☐ | ☐ |
| 3. | … avez-vous joué à des jeux pour oublier votre vie quotidienne ? | ☐ | ☐ | ☐ | ☐ | ☐ |
| 4. | … est-ce que d'autres personnes de votre entourage ont essayé, sans succès, de vous faire réduire le temps que vous passez à jouer ? | ☐ | ☐ | ☐ | ☐ | ☐ |
| 5. | … vous êtes-vous senti mal quand vous ne pouviez pas jouer ? | ☐ | ☐ | ☐ | ☐ | ☐ |
| 6. | … avez-vous eu des conflits avec des membres de votre entourage (famille, amis) en raison du temps que vous passez à jouer ? | ☐ | ☐ | ☐ | ☐ | ☐ |
| 7. | … avez-vous négligé d'autres activités importantes (cours, travail, sport) pour jouer ? | ☐ | ☐ | ☐ | ☐ | ☐ |

**German**

Wie oft in den LETZTEN 6 MONATEN …

| | *Kreuzen Sie in jeder Zeile ein Kästchen an.* | **Nie** *1* | **Selten** *2* | **Manchmal** *3* | **Oft** *4* | **Sehr oft** *5* |
|---|---|---|---|---|---|---|
| 1. | …haben Sie den ganzen Tag ans 'gamen' gedacht? | ☐ | ☐ | ☐ | ☐ | ☐ |
| 2. | …haben Sie länger 'gegamet' als ursprünglich beabsichtigt? | ☐ | ☐ | ☐ | ☐ | ☐ |
| 3. | …haben Sie 'gegamet', um Ihren Alltag zu vergessen? | ☐ | ☐ | ☐ | ☐ | ☐ |
| 4. | … haben andere vergeblich versucht, Sie dazu zu bringen, weniger zu 'gamen'? | ☐ | ☐ | ☐ | ☐ | ☐ |
| 5. | … haben Sie sich schlecht gefühlt, wenn Sie nicht 'gamen' konnten? | ☐ | ☐ | ☐ | ☐ | ☐ |
| 6. | ... haben Sie Streit gehabt mit anderen (z.B. Familie, Freunde) wegen der Zeit, die Sie mit 'gamen' verbrachten? | ☐ | ☐ | ☐ | ☐ | ☐ |
| 7. | ...haben Sie wichtige Aktivitäten (z.B. Schule, Arbeit, Sport) vernachlässigt, um 'gamen' zu können? | ☐ | ☐ | ☐ | ☐ | ☐ |

### *S1.3 Internet*

The study assessed symptoms of problematic internet use, as conceptualized by the Compulsive Internet Use Scale (CIUS; Meerkerk et al., 2010; Meerkerk et al., 2009). The scale was developed by Meerkerk et al. (2009) based on the DSM-IV criteria for pathological gambling (APA, 1994) and criteria for behavioral addictions elaborated by Griffiths (1999, 2005).

Validated French and German versions of the scale were used, as reported by Khazaal et al. (2012) and Guertler et al. (2014), respectively.

**English**
How often …

| | *Tick one box in each row.* | **Never** *0* | **Rarely** *1* | **Some-times** *2* | **Often** *3* | **Very often** *4* |
|---|---|---|---|---|---|---|
| 1. | … do you find it difficult to stop using the Internet when you are online? | ☐ | ☐ | ☐ | ☐ | ☐ |
| 2. | … do you continue to use the Internet despite your intention to stop? | ☐ | ☐ | ☐ | ☐ | ☐ |
| 3. | … do others (e.g. partner, children, parents) say you should use the Internet less? | ☐ | ☐ | ☐ | ☐ | ☐ |
| 4. | … do you prefer to use the Internet instead of spending time with others (e.g. partner, children, parents)? | ☐ | ☐ | ☐ | ☐ | ☐ |
| 5. | … are you short of sleep because of the Internet? | ☐ | ☐ | ☐ | ☐ | ☐ |
| 6. | … do you think about the Internet, even when not online? | ☐ | ☐ | ☐ | ☐ | ☐ |
| 7. | … do you look forward to your next Internet session? | ☐ | ☐ | ☐ | ☐ | ☐ |
| 8. | … do you think you should use the Internet less often? | ☐ | ☐ | ☐ | ☐ | ☐ |
| 9. | … have you unsuccessfully tried to spend less time on the Internet? | ☐ | ☐ | ☐ | ☐ | ☐ |
| 10. | … do you rush through your (home) work in order to go on the Internet? | ☐ | ☐ | ☐ | ☐ | ☐ |
| 11. | … do you neglect your daily obligations (work, school, or family life) because you prefer to go on the Internet? | ☐ | ☐ | ☐ | ☐ | ☐ |
| 12. | … do you go on the Internet when you are feeling down? | ☐ | ☐ | ☐ | ☐ | ☐ |
| 13. | … do you use the Internet to escape from your sorrows or get relief from negative feelings? | ☐ | ☐ | ☐ | ☐ | ☐ |
| 14. | … do you feel restless, frustrated, or irritated when you cannot use the Internet? | ☐ | ☐ | ☐ | ☐ | ☐ |

**French**
A QUELLE FREQUENCE …

| | *Cochez une case par ligne* | Jamais *0* | Rarement *1* | Parfois *2* | Souvent *3* | Très souvent *4* |
|---|---|---|---|---|---|---|
| 1. | … trouvez-vous difficile d'arrêter d'utiliser Internet une fois que vous êtes en ligne ? | ☐ | ☐ | ☐ | ☐ | ☐ |
| 2. | … continuez-vous d'utiliser Internet malgré votre intention d'arrêter ? | ☐ | ☐ | ☐ | ☐ | ☐ |
| 3. | … d'autres personnes (ex : partenaire, enfants, parents) vous disent-elles que vous devriez moins utiliser Internet ? | ☐ | ☐ | ☐ | ☐ | ☐ |
| 4. | … préférez-vous aller sur Internet au lieu de passer du temps avec d'autres personnes (partenaires, enfants, parents) ? | ☐ | ☐ | ☐ | ☐ | ☐ |
| 5. | … vous arrive-t-il de manquer de sommeil à cause d'Internet ? | ☐ | ☐ | ☐ | ☐ | ☐ |
| 6. | … pensez-vous à Internet même quand vous n'êtes pas en ligne ? | ☐ | ☐ | ☐ | ☐ | ☐ |
| 7. | … vous réjouissez-vous de votre prochaine connexion à Internet ? | ☐ | ☐ | ☐ | ☐ | ☐ |
| 8. | … pensez-vous que vous devriez utiliser Internet moins souvent ? | ☐ | ☐ | ☐ | ☐ | ☐ |
| 9. | … avez-vous essayé sans succès de passer moins de temps sur Internet ? | ☐ | ☐ | ☐ | ☐ | ☐ |
| 10. | … vous arrive-t-il de vous dépêcher de faire quelque chose (travail, ménages…) pour aller sur Internet ? | ☐ | ☐ | ☐ | ☐ | ☐ |
| 11. | … négligez-vous vos obligations quotidiennes (travail, école, ou vie familiale) parce que vous préférez aller sur Internet ? | ☐ | ☐ | ☐ | ☐ | ☐ |
| 12. | … allez-vous sur Internet quand vous n'avez pas le moral ? | ☐ | ☐ | ☐ | ☐ | ☐ |
| 13. | … utilisez-vous Internet pour fuir vos soucis ou vous soulager d'un sentiment négatif ? | ☐ | ☐ | ☐ | ☐ | ☐ |
| 14. | … vous sentez-vous agité, frustré ou irrité lorsque vous ne pouvez pas utiliser Internet ? | ☐ | ☐ | ☐ | ☐ | ☐ |

**German**
Wie häufig …

| | *Kreuzen Sie in jeder Zeile ein Kästchen an.* | Nie *0* | Selten *1* | Manchmal *2* | Oft *3* | Sehr oft *4* |
|---|---|---|---|---|---|---|
| 1. | … finden Sie es schwierig, mit dem Internetgebrauch aufzuhören, wenn Sie online sind? | ☐ | ☐ | ☐ | ☐ | ☐ |
| 2. | … setzen Sie Ihren Internetgebrauch fort, obwohl Sie eigentlich aufhören wollten? | ☐ | ☐ | ☐ | ☐ | ☐ |
| 3. | … sagen Ihnen andere Menschen (z.B. Partner, Kinder, Eltern, Freunde), dass Sie das Internet weniger nutzen sollten? | ☐ | ☐ | ☐ | ☐ | ☐ |
| 4. | … bevorzugen Sie das Internet, statt Zeit mit anderen zu verbringen (z.B. Partner, Kinder, Eltern, Freunde)? | ☐ | ☐ | ☐ | ☐ | ☐ |
| 5. | … schlafen Sie zu wenig wegen des Internets? | ☐ | ☐ | ☐ | ☐ | ☐ |
| 6. | … denken Sie an das Internet, auch wenn Sie gerade nicht online sind? | ☐ | ☐ | ☐ | ☐ | ☐ |
| 7. | … freuen Sie sich bereits auf Ihre nächste Internetsitzung? | ☐ | ☐ | ☐ | ☐ | ☐ |
| 8. | … denken Sie darüber nach, dass Sie weniger Zeit im Internet verbringen sollten? | ☐ | ☐ | ☐ | ☐ | ☐ |
| 9. | … haben Sie erfolglos versucht, weniger Zeit im Internet zu verbringen? | ☐ | ☐ | ☐ | ☐ | ☐ |
| 10. | … erledigen Sie Ihre Aufgaben (zu Hause oder auf der Arbeit) hastig, damit Sie früher ins Internet können? | ☐ | ☐ | ☐ | ☐ | ☐ |
| 11. | … vernachlässigen Sie Ihre Alltagsverpflichtungen (Arbeit, Schule, Familienleben), weil Sie lieber ins Internet gehen? | ☐ | ☐ | ☐ | ☐ | ☐ |
| 12. | … gehen Sie ins Internet, wenn Sie sich niedergeschlagen fühlen? | ☐ | ☐ | ☐ | ☐ | ☐ |
| 13. | … nutzen Sie das Internet, um Ihren Sorgen zu entkommen oder um sich von einer negativen Stimmung zu entlasten? | ☐ | ☐ | ☐ | ☐ | ☐ |
| 14. | … fühlen Sie sich unruhig, frustriert oder gereizt, wenn Sie das Internet nicht nutzen können? | ☐ | ☐ | ☐ | ☐ | ☐ |

### *S1.4 Pornography*

The study assessed symptoms of internet pornography use disorder, as conceptualized by the Online Sexual Compulsivity subscale of the Internet Sex Screening Test (ISST; Carnes et al., 2009; Delmonico & Miller, 2003). This subscale measures excessive or uncontrollable engagement in online sexual activities, which may result in distress or negative consequences in personal, social, or occupational domains (Delmonico & Miller, 2003).

The French and German versions were translated by bilingual members of the research team using the forward-backward translation method.

**English**

Please indicate to what extent each of the following statements below apply to your situation. Check « true » if the statements apply to your situation DURING THE PAST 12 MONTHS. Check « false » if the statements do not apply to your situation DURING THE PAST 12 MONTHS.

| | *Tick one box in each row* | **Yes** *1* | **No** *0* |
|---|---|---|---|
| 1. | Internet sex has sometimes interfered with certain aspects of my life. | ☐ | ☐ |
| 2. | I have made promises to myself to stop using the Internet for sexual purposes. | ☐ | ☐ |
| 3. | I sometimes use cybersex as a reward for accomplishing something (e.g. finish a project, stressful day, etc.) | ☐ | ☐ |
| 4. | When I am unable to access sexual information online, I feel anxious, angry, or disappointed. | ☐ | ☐ |
| 5. | I have punished myself when I use the Internet for sexual purposes (e.g. time-out from computer, cancel Internet subscription, etc.) | ☐ | ☐ |
| 6. | I believe I am an Internet sex addict. | ☐ | ☐ |

**French**

Pouvez-vous indiquer dans quelle mesure chacune des affirmations ci-dessous s'appliquent à votre situation. Cochez « vrai » si les affirmations correspondent à votre situation AU COURS DES 12 DERNIERS MOIS. Cochez « faux » si elles ne correspondent pas à votre situation AU COURS DES 12 DERNIERS MOIS.

| | *Cochez une case par ligne.* | **Vrai** | **Faux** |
|---|---|---|---|
| | | *1* | *0* |
| 1. | Le sexe sur Internet a parfois interféré avec certains aspects de ma vie. | ☐ | ☐ |
| 2. | Je me suis promis d'arrêter d'utiliser Internet à des fins sexuelles. | ☐ | ☐ |
| 3. | J'utilise parfois le cybersexe comme une récompense pour avoir accompli quelque chose (par ex. finir un projet, après un jour stressant, etc.). | ☐ | ☐ |
| 4. | Lorsque je ne peux pas accéder à de l'information sexuelle en ligne, je me sens anxieux, en colère, ou déçu. | ☐ | ☐ |
| 5. | Après avoir utilisé Internet à des fins sexuelles, je me punis de l'avoir fait (par ex. ne plus utiliser mon ordinateur, annuler mon abonnement à Internet, etc.). | ☐ | ☐ |
| 6. | Je crois que je suis un accro au sexe sur Internet. | ☐ | ☐ |

**German**

Geben Sie bitte an, was AM EHESTEN auf Ihr Verhalten zutrifft. Fragen Sie sich, ob die unten beschriebene Situation in den LETZTEN 12 MONATEN vorgekommen ist und antworten Sie mit „richtig" oder „falsch".

| | *Kreuzen Sie in jeder Zeile ein Kästchen an.* | **Richtig** | **Falsch** |
|---|---|---|---|
| | | *1* | *0* |
| 1. | Internetsex hat manchmal andere Bereiche meines Lebens beeinträchtigt. | ☐ | ☐ |
| 2. | Ich habe mir schon mal geschworen, aufzuhören, das Internet für sexuelle Zwecke zu benutzen. | ☐ | ☐ |
| 3. | Ich benutze manchmal Internetsex als Belohnung dafür, dass ich andere Dinge geschafft habe (z.B. ein Projekt beendet zu haben, einen stressigen Tag hinter mich gebracht zu haben). | ☐ | ☐ |
| 4. | Wenn ich nicht im Internet auf Sexseiten gehen kann, fühle ich mich ängstlich, wütend oder enttäuscht. | ☐ | ☐ |
| 5. | Ich habe mich dafür bestraft, wenn ich das Internet für sexuelle Zwecke benutzt habe (z.B. indem ich eine Auszeit vom Computer genommen, oder den Internetzugang gestrichen habe). | ☐ | ☐ |
| 6. | Ich glaube ich bin internetsexsüchtig. | ☐ | ☐ |

### S1.5 Smartphone

The study assessed symptoms of problematic smartphone use, as conceptualized by the Short Version of the Smartphone Addiction Scale (SAS-SV; Kwon, Kim, et al., 2013). The SAS-SV is derived from the original Smartphone Addiction Scale (SAS; Kwon, Lee, et al., 2013) and draws on the core components of addiction (Griffiths, 2005), considering six factors: daily-life disturbance, positive anticipation, withdrawal, cyberspace-oriented relationships, overuse, and tolerance.

The German version has been translated and validated by Haug et al. (2015). The French version was translated by bilingual members of the research team using the forward-backward translation method.

**English**
In relation with your smartphone, please indicate to what extent you agree/disagree with the following statements?

| | *Tick one box in each row.* | Strongly disagree | Disagree | Not completely agree | Somewhat agree | Agree | Strongly agree |
|---|---|---|---|---|---|---|---|
| | | *0* | *1* | *2* | *3* | *4* | *5* |
| 1. | I miss planned work due to smartphone use | ☐ | ☐ | ☐ | ☐ | ☐ | ☐ |
| 2. | I have a hard time concentrating in class, while doing assignments, or while working due to smartphone use | ☐ | ☐ | ☐ | ☐ | ☐ | ☐ |
| 3. | I feel pain in the wrists or at the back of the neck while using a smartphone | ☐ | ☐ | ☐ | ☐ | ☐ | ☐ |
| 4. | I won't be able to stand not having a smartphone | ☐ | ☐ | ☐ | ☐ | ☐ | ☐ |
| 5. | I feel impatient and fretful when I am not holding my smartphone | ☐ | ☐ | ☐ | ☐ | ☐ | ☐ |
| 6. | I have my smartphone in my mind even when I am not using it | ☐ | ☐ | ☐ | ☐ | ☐ | ☐ |
| 7. | I will never give up using my smartphone even when my daily life is already greatly affected by it. | ☐ | ☐ | ☐ | ☐ | ☐ | ☐ |
| 8. | I constantly check my smartphone so as not to miss conversations between other people on Twitter or Facebook | ☐ | ☐ | ☐ | ☐ | ☐ | ☐ |
| 9. | I use my smartphone longer than I had intended | ☐ | ☐ | ☐ | ☐ | ☐ | ☐ |
| 10. | The people around me tell me that I use my smartphone too much | ☐ | ☐ | ☐ | ☐ | ☐ | ☐ |

**French**
A quel point êtes-vous d'accord avec les propositions suivantes ?

| | *Cochez une case par ligne* | Pas du tout d'accord *0* | Pas d'accord *1* | Pas tout à fait d'accord *2* | Un peu d'accord *3* | D'accord *4* | Tout à fait d'accord *5* |
|---|---|---|---|---|---|---|---|
| 1. | En raison de l'utilisation de mon smartphone, il m'est difficile d'accomplir le travail planifié. | ☐ | ☐ | ☐ | ☐ | ☐ | ☐ |
| 2. | En raison de l'utilisation de mon smartphone, j'ai du mal à me concentrer en classe, en faisant mes devoirs, ou en travaillant. | ☐ | ☐ | ☐ | ☐ | ☐ | ☐ |
| 3. | En raison de l'utilisation de mon smartphone, je ressens de la douleur à la nuque ou aux poignets. | ☐ | ☐ | ☐ | ☐ | ☐ | ☐ |
| 4. | Je ne pourrais pas supporter de ne pas avoir de smartphone. | ☐ | ☐ | ☐ | ☐ | ☐ | ☐ |
| 5. | Lorsque je n'ai pas mon smartphone à portée de main, je suis impatient et agité. | ☐ | ☐ | ☐ | ☐ | ☐ | ☐ |
| 6. | Je pense constamment à mon smartphone, même lorsque je ne l'utilise pas. | ☐ | ☐ | ☐ | ☐ | ☐ | ☐ |
| 7. | Je ne renoncerai jamais à utiliser mon smartphone, même si cela affecte beaucoup ma vie quotidienne. | ☐ | ☐ | ☐ | ☐ | ☐ | ☐ |
| 8. | Pour ne manquer aucune nouvelle, je dois continuellement contrôler mon smartphone (sur Twitter ou Facebook). | ☐ | ☐ | ☐ | ☐ | ☐ | ☐ |
| 9. | Quand j'utilise mon smartphone, je l'utilise plus longtemps que prévu initialement. | ☐ | ☐ | ☐ | ☐ | ☐ | ☐ |
| 10. | Les personnes autour de moi disent que j'utilise trop mon smartphone. | ☐ | ☐ | ☐ | ☐ | ☐ | ☐ |

**German**

Geben Sie bitte an, wie stark die folgenden Aussagen auf Sie zutreffen.

| | *Kreuzen Sie in jeder Zeile ein Kästchen an.* | **Gar nicht zutreffend** *0* | **Nicht zutreffend** *1* | **Eher nicht zutreffend** *2* | **Ein wenig zutreffend** *3* | **Zutreffend** *4* | **Sehr zutreffend** *5* |
|---|---|---|---|---|---|---|---|
| 1. | Wegen des Gebrauchs meines Smartphones fällt es mir schwer, geplante Aufgaben zu erledigen. | ☐ | ☐ | ☐ | ☐ | ☐ | ☐ |
| 2. | Wegen des Gebrauchs meines Smartphones fällt es mir schwer, mich auf den Unterricht in der Schule oder auf meine Arbeit zu konzentrieren. | ☐ | ☐ | ☐ | ☐ | ☐ | ☐ |
| 3. | Ich spüre Schmerzen im Nacken oder im Handgelenk aufgrund des Gebrauchs meines Smartphones. | ☐ | ☐ | ☐ | ☐ | ☐ | ☐ |
| 4. | Ich könnte es nicht ertragen, kein Smartphone zu besitzen. | ☐ | ☐ | ☐ | ☐ | ☐ | ☐ |
| 5. | Wenn ich mein Smartphone nicht zur Hand habe, bin ich unruhig und gereizt. | ☐ | ☐ | ☐ | ☐ | ☐ | ☐ |
| 6. | Ich muss ständig an mein Smartphone denken, sogar dann, wenn ich es nicht benutze. | ☐ | ☐ | ☐ | ☐ | ☐ | ☐ |
| 7. | Auch wenn durch meinen Gebrauch des Smartphones schon Probleme im Alltag aufgetreten sind, kann ich nicht darauf verzichten. | ☐ | ☐ | ☐ | ☐ | ☐ | ☐ |
| 8. | Um keine Neuigkeiten (z.B. auf Twitter, Facebook) zu verpassen, muss ich ständig mein Smartphone kontrollieren. | ☐ | ☐ | ☐ | ☐ | ☐ | ☐ |
| 9. | Wenn ich einmal damit angefangen habe, beschäftige ich mich länger mit meinem Smartphone als beabsichtigt. | ☐ | ☐ | ☐ | ☐ | ☐ | ☐ |
| 10. | Die Menschen in meinem Umfeld sagen mir, dass ich zu viel Zeit mit meinem Smartphone verbringe. | ☐ | ☐ | ☐ | ☐ | ☐ | ☐ |

### *S1.6 Work*

The study assessed symptoms of work addiction, as conceptualized by the Bergen Work Addiction Scale (BWAS; Andreassen et al., 2012). The BWAS items align with the six components of addiction by Griffiths (2005)—salience, mood modification, tolerance, withdrawal, conflict, and relapse—along with an additional item addressing negative consequences or problems.

The French and German versions were translated by bilingual members of the research team using the forward-backward translation method.

**English**

Below you find seven questions related to your work/job/study. How often during the last 12 months have you …

| | *Tick one box in each row.* | **Never** *1* | **Rarely** *2* | **Some-times** *3* | **Often** *4* | **Always** *5* |
|---|---|---|---|---|---|---|
| 1. | …thought of how you could free up more time to work? | ☐ | ☐ | ☐ | ☐ | ☐ |
| 2. | …spent much more time working than initially intended? | ☐ | ☐ | ☐ | ☐ | ☐ |
| 3. | …worked in order to reduce feelings of guilt, anxiety, helplessness a d depression? | ☐ | ☐ | ☐ | ☐ | ☐ |
| 4. | …been told by others to cut down on work without listening to them? | ☐ | ☐ | ☐ | ☐ | ☐ |
| 5. | …become stressed if you have been prohibited from working? | ☐ | ☐ | ☐ | ☐ | ☐ |
| 6. | …deprioritized hobbies, leisure activities, and exercise because of your work? | ☐ | ☐ | ☐ | ☐ | ☐ |
| 7. | … worked so much that it has negatively influenced your health? | ☐ | ☐ | ☐ | ☐ | ☐ |

**French**

Pensez à votre (vos) travail/études/apprentissage, au cours des 12 derniers mois, à quelle fréquence …

| | *Cochez une case par ligne.* | **Jamais** *1* | **Rarement** *2* | **Parfois** *3* | **Souvent** *4* | **Toujours** *5* |
|---|---|---|---|---|---|---|
| 1. | …avez-vous réfléchi à la manière dont vous pourriez libérer plus de temps pour travailler ? | ☐ | ☐ | ☐ | ☐ | ☐ |
| 2. | …avez-vous passé plus de temps à travailler que prévu initialement ? | ☐ | ☐ | ☐ | ☐ | ☐ |
| 3. | …avez-vous travaillé afin de réduire des sentiments de culpabilité, d'anxiété, d'impuissance ou de dépression ? | ☐ | ☐ | ☐ | ☐ | ☐ |
| 4. | …des personnes vous ont suggéré de travailler moins sans que vous les écoutiez ? | ☐ | ☐ | ☐ | ☐ | ☐ |
| 5. | …êtes-vous devenu stressé si vous ne pouviez pas travailler ? | ☐ | ☐ | ☐ | ☐ | ☐ |
| 6. | …avez-vous réduit la priorité de vos hobbies, loisirs, et activités physiques (exercice) à cause de votre travail ? | ☐ | ☐ | ☐ | ☐ | ☐ |
| 7. | …avez-vous travaillé tellement que cela a nuit à votre santé ? | ☐ | ☐ | ☐ | ☐ | ☐ |

**German**

Denken Sie an Ihre Arbeit, an Ihr Studium oder an Ihre Ausbildung. Wie häufig haben Sie in den letzten 12 Monaten …

| | *Kreuzen Sie ein Kästchen pro Zeile an.* | **Nie** *1* | **Selten** *2* | **Manchmal** *3* | **Oft** *4* | **Immer** *5* |
|---|---|---|---|---|---|---|
| 1. | ... darüber nachgedacht, wie Sie mehr Zeit zum Arbeiten finden können? | ☐ | ☐ | ☐ | ☐ | ☐ |
| 2. | ...viel mehr gearbeitet, als ursprünglich beabsichtigt? | ☐ | ☐ | ☐ | ☐ | ☐ |
| 3. | ...gearbeitet, um Gefühle wie Schuld, Hilflosigkeit, Depressionen oder Angst zu reduzieren? | ☐ | ☐ | ☐ | ☐ | ☐ |
| 4. | ... von anderen gesagt bekommen, dass Sie weniger arbeiten sollten, haben aber nicht auf sie gehört? | ☐ | ☐ | ☐ | ☐ | ☐ |
| 5. | ... sich gestresst gefühlt, wenn Sie nicht arbeiten konnten? | ☐ | ☐ | ☐ | ☐ | ☐ |
| 6. | ... Hobbies, Freizeitaktivitäten und sportliche Betätigungen wegen Ihrer Arbeit vernachlässigt? | ☐ | ☐ | ☐ | ☐ | ☐ |
| 7. | ... so viel gearbeitet, dass Ihre Gesundheit darunter gelitten hat? | ☐ | ☐ | ☐ | ☐ | ☐ |

### *S1.7 Alcohol use disorder (AUD)*

Symptoms of alcohol use disorder were assessed using the eleven DSM-5 criteria (APA, 2013). Most items were taken from Knight et al. (2002), with an additional item on craving taken from Grant et al. (2003). The DSM-5 criterion assessing "engagement in physically hazardous situations" was measured with two items: item 2 (driving under the influence) and item 3 (engaging in activities with an increased risk of injury). This criterion was coded as present if at least one of the two items was endorsed (Knight et al., 2002).

The French and German versions were translated by bilingual members of the research team using the forward-backward translation method.

**English**
Think of the PAST 12 MONTHS and choose one answer in each row.

| | *Tick one box in each row* | **Yes** *1* | **No** *0* |
|---|---|---|---|
| 1. | …has your drinking alcohol caused you **more than once** to miss a class, work or to fail to look after your family at home? | ☐ | ☐ |
| 2. | ...did you **more than once** drive a car or another vehicle (such as a bicycle, motorcycle or moped) shortly after you had had several drinks with alcohol? | ☐ | ☐ |
| 3. | …did you find yourself **more than once** in a situation that increased your chances of getting injured (using machines, walking or doing sport in a dangerous area or around heavy traffic) after you had been drinking too much alcohol? | ☐ | ☐ |
| 4. | …did you resume **your drinking habits** even though your drinking had caused **problems with your partner, friend or acquaintances**? | ☐ | ☐ |
| 5. | …did you find you needed **a lot more alcohol** to become high or drunk than you used to? | ☐ | ☐ |
| 6. | …did you **start feeling nervous or shaky** for a full day or more after you had cut down on your drinking? | ☐ | ☐ |
| 7. | …did you often find yourself **drinking more and for longer periods of time** than you intended? | ☐ | ☐ |
| 8. | …did you try **to cut down on your drinking**, but couldn't? | ☐ | ☐ |
| 9. | …did you find yourself **spending a great deal of time** obtaining, using, or recovering from the effects of alcohol? | ☐ | ☐ |
| 10. | …did you **give up** activities you care about (e.g. **school, work or being with friends and family**) because of your drinking? | ☐ | ☐ |
| 11. | …did you continue drinking even though you were aware that alcohol had repeatedly caused you **anxiety, depression or health problems**? | ☐ | ☐ |
| 12. | …have you had such a **strong desire or urge to drink** that you could not help drinking? | ☐ | ☐ |

**French**
AU COURS DES 12 DERNIERS MOIS, vous est-il arrivé ...

| | *Cochez une case par ligne.* | **Oui** *1* | **Non** *0* |
|---|---|---|---|
| 1. | …de négliger **plus d'une fois** des tâches importantes à l'école, au travail, ou à la maison dans le cadre de votre famille parce que vous aviez consommé de l'alcool ou parce que vous vous sentiez mal après en avoir consommé ? | ☐ | ☐ |
| 2. | …de **conduire plus d'une fois** un **véhicule** (p.ex. voiture, vélo, vélomoteur, scooter ou moto) après avoir bu quelques verres ? | ☐ | ☐ |
| 3. | …de vous être trouvé **plus d'une fois** dans une situation dans laquelle vous auriez pu vous blesser après avoir trop bu (p.ex. avec des outils au travail ou sur des machines, en faisant du sport ou d'autres situations dangereuses telles que marcher au milieu de la route) ? | ☐ | ☐ |
| 4. | …d'avoir repris **vos habitudes de consommation** d'alcool alors que celles-ci avaient généré des **problèmes avec votre partenaire, un(e) ami(e) ou connaissance** ? | ☐ | ☐ |
| 5. | …d'avoir l'impression que vous devez consommer **beaucoup plus** d'alcool qu'auparavant pour en ressentir les effets ou être saoul ? | ☐ | ☐ |
| 6. | …**d'avoir tremblé ou de vous être senti nerveux** pendant toute une journée ou plus après avoir arrêté votre consommation ? | ☐ | ☐ |
| 7. | …d'avoir **bu davantage et pendant plus longtemps** que vous ne l'aviez **prévu** ? | ☐ | ☐ |
| 8. | …d'avoir essayé **de réduire votre consommation**, mais sans succès ? | ☐ | ☐ |
| 9. | …**d'avoir passé beaucoup de temps** soit à vous saouler, à être saoul, ou à dessaouler ? | ☐ | ☐ |
| 10. | …**d'avoir abandonné** des activités importantes telles **que l'école, le travail ou les rencontres entre amis ou avec la famille** à cause de votre consommation d'alcool ? | ☐ | ☐ |
| 11. | …d'avoir continué votre consommation d'alcool bien que vous saviez qu'elle avait généré des problèmes récurrents de santé ou des problèmes psychiques tels **que l'anxiété ou la dépression** ? | ☐ | ☐ |
| 12. | …d'avoir ressenti **un besoin ou un désir si fort** que vous ne pouviez pas faire autrement que de boire de l'alcool ? | ☐ | ☐ |

**German**
Ist es in den LETZTEN 12 MONATEN vorgekommen ...

| | *Kreuzen Sie in jeder Zeile ein Kästchen an.* | **Ja** *1* | **Nein** *0* |
|---|---|---|---|
| 1. | ... dass Sie wegen ihres Alkoholkonsums oder weil es ihnen danach nicht so gut ging, **mehr als einmal** wichtige Verpflichtungen in der Schule oder auf der Arbeit oder auch zu Hause mit ihrer Familie vernachlässigt haben? | ☐ | ☐ |
| 2. | ...dass Sie **mehr als einmal** ein **Fahrzeug** (z. B. ein Fahrrad, ein Moped, einen Roller oder ein Motorrad) **gelenkt** haben, nachdem Sie mehrere Gläser getrunken hatten? | ☐ | ☐ |
| 3. | …dass Sie sich **mehr als einmal** in Situationen befunden haben, bei denen Sie sich hätten verletzen können (z.B. beim Arbeiten mit Werkzeugen oder an Maschinen, beim Sport oder in anderen gefährlichen Situationen z.B. als Fussgänger im Strassenverkehr) nachdem Sie zu viel getrunken haben? | ☐ | ☐ |
| 4. | …dass Sie zu Ihrem **gewohnten Alkoholkonsum** zurückgekehrt sind, obwohl dieser **Probleme mit dem Partner/der Partnerin**, einem **Freund/einer Freundin** oder mit einer **Bezugsperson** verursacht hat? | ☐ | ☐ |
| 5. | … dass Sie das Gefühl hatten, dass Sie **viel mehr** als früher Alkohol konsumieren müssen, um angetrunken oder betrunken zu werden? | ☐ | ☐ |
| 6. | ... dass Sie sich während eines ganzen Tages oder länger **zittrig oder nervös** gefühlt haben, nachdem Sie Ihren Alkoholkonsum gestoppt haben? | ☐ | ☐ |
| 7. | ... dass Sie **länger und mehr** getrunken haben, **als Sie dies geplant hatten**? | ☐ | ☐ |
| 8. | … dass Sie versucht haben Ihren **Konsum zu reduzieren**, es aber nicht schafften? | ☐ | ☐ |
| 9. | ... dass Sie **viel Zeit damit verbracht** haben, sich einen Rausch anzutrinken, berauscht zu sein, oder damit, sich vom Rausch zu erholen? | ☐ | ☐ |
| 10. | ... dass Sie wegen Ihres Alkoholkonsums wichtige Aktivitäten wie **Schule, Arbeit oder sich mit Freunden und Familie treffen aufgegeben** haben? | ☐ | ☐ |
| 11. | ... dass Sie Ihren Alkoholkonsum fortgesetzt haben, obwohl Sie wussten, dass er bei Ihnen wiederholt gesundheitliche Probleme oder psychische Probleme wie **Ängste oder Depressionen** verursacht hatte? | ☐ | ☐ |
| 12. | ... dass Sie so ein **starkes Verlangen oder einen starken Drang** danach verspürten, dass Sie nicht anders konnten, als Alkohol zu trinken? | ☐ | ☐ |

### *S1.8 Cannabis use disorder (CUD)*

Symptoms of cannabis use disorder were assessed using the Revised Cannabis Use Disorder Identification Test (CUDIT-R; Annaheim et al., 2010), a revised version of the Cannabis Use Disorder Identification Test (CUDIT; Adamson & Sellman, 2003). The revision, as described by Annaheim et al. (2010), was guided by the working definition of problematic cannabis use adopted from the European Monitoring Centre for Drugs and Drug Addiction (EMCDDA), which defines it as a pattern of use that leads to negative social or health-related consequences for the user or their environment (Beck & Legleye, 2008). Compared to the original CUDIT (Adamson & Sellman, 2003), the CUDIT-R omits three items—usual number of hours being stoned, feelings of guilt/remorse, and injuries—and replaces them with items assessing missed leisure activities, difficulties at school or work, and motives for cannabis use (Annaheim et al., 2010).

The original CUDIT was developed by adapting the Alcohol Use Disorders Identification Test (AUDIT; Saunders et al., 1993) to assess cannabis use and related problems. The AUDIT was constructed from a pool of items drawn from existing alcohol screening instruments, with item selection guided by the World Health Organization's conceptual definitions of hazardous and harmful alcohol use (Saunders et al., 1993; World Health Organization, 1992). In World Health Organization terminology, hazardous use refers to consumption that increases the risk of physical or psychological harm (Edwards et al., 1981), while harmful use is defined in ICD-10 as the presence of actual physical or psychological complications (World Health Organization, 1992).

The German version was validated by Annaheim et al. (2010). The French version was translated by bilingual members of the research team using the forward-backward translation method.

**English**
IN THE PAST 12 MONTHS, how often did you usually smoke cannabis?

| | Once a month or less<br>*0* | 2 to 4 times a month<br>*1* | 2 à 3 times a week<br>*2* | 4 à 5 times a week<br>*3* | Every day or nearly every day<br>*4* |
|---|---|---|---|---|---|
| 1. | ☐ | ☐ | ☐ | ☐ | ☐ |

Now think of the PAST 12 MONTHS:

| | *Tick one box in each row.* | Never<br>*0* | Less than once a month<br>*1* | Once a month<br>*2* | Once a week<br>*3* | Daily or nearly every day<br>*4* |
|---|---|---|---|---|---|---|
| 2. | How often have you felt "stoned" for 6 or more hours? | ☐ | ☐ | ☐ | ☐ | ☐ |
| 3. | How often have you found that you were not able to stop using cannabis once you had started? | ☐ | ☐ | ☐ | ☐ | ☐ |
| 4. | How often have you failed to do what was normally expected from you because of using cannabis? | ☐ | ☐ | ☐ | ☐ | ☐ |
| 5. | How often have you been in the need of cannabis in the morning to get yourself going after a heavy cannabis intake the day before? | ☐ | ☐ | ☐ | ☐ | ☐ |
| 6. | How often have you had a problem with your memory or concentration after using cannabis? | ☐ | ☐ | ☐ | ☐ | ☐ |
| 7. | How often have you refrained from taking part in leisure time activities that you originally wanted to do, e.g. going out, sports, hobbies, etc., because of using cannabis? | ☐ | ☐ | ☐ | ☐ | ☐ |
| 8. | How often have you had difficulties at work or school, because of using cannabis? | ☐ | ☐ | ☐ | ☐ | ☐ |

Has a relative, friend or a doctor or other health worker been concerned about your use of cannabis or suggested you cut down OVER THE PAST 12 MONTHS?

| | No<br>*0* | Yes<br>*4* |
|---|---|---|
| 9. | ☐ | ☐ |

Which of the following statements best fits your personal situation?

| | I smoke cannabis for fun, because it's something special.<br>*0* | I smoke cannabis out of habit, because it's part of my daily life.<br>*4* |
|---|---|---|
| 10. | ☐ | ☐ |

**French**

En pensant AUX 12 DERNIERS MOIS, à quelle fréquence preniez-vous habituellement du cannabis ?

| | 1 fois par mois ou moins souvent<br>*0* | 2 à 4 fois par mois<br>*1* | 2 à 3 fois par semaine<br>*2* | 4 à 5 fois par semaine<br>*3* | Tous les jours ou presque tous les jours<br>*4* |
|---|---|---|---|---|---|
| 1. | ☐ | ☐ | ☐ | ☐ | ☐ |

En pensant AUX 12 DERNIERS MOIS, veuillez répondre aux questions suivantes:

| | *Cochez une case par ligne.* | Jamais<br>*0* | Moins d'une fois par mois<br>*1* | Une fois par mois<br>*2* | Une fois par semaine<br>*3* | Chaque jour ou presque<br>*4* |
|---|---|---|---|---|---|---|
| 2. | Combien de fois avez-vous été "pété" pendant au moins 6 heures après avoir pris du cannabis ? | ☐ | ☐ | ☐ | ☐ | ☐ |
| 3. | Combien de fois avez-vous eu l'impression de ne plus pouvoir vous arrêter de prendre du cannabis ? | ☐ | ☐ | ☐ | ☐ | ☐ |
| 4. | Combien de fois n'étiez-vous plus en mesure de faire ce que l'on attend normalement de vous à cause de la prise de cannabis ? | ☐ | ☐ | ☐ | ☐ | ☐ |
| 5. | Combien de fois avez-vous pris du cannabis le matin pour être à nouveau en forme après une forte consommation de cannabis la veille ? | ☐ | ☐ | ☐ | ☐ | ☐ |
| 6. | Combien de fois avez-vous eu des problèmes de mémoire ou de concentration à cause de votre consommation de cannabis ? | ☐ | ☐ | ☐ | ☐ | ☐ |
| 7. | Combien de fois avez-vous renoncé, durant vos loisirs, à une activité que vous auriez en fait voulu faire - par exemple une sortie, du sport, un hobby, etc. - à cause de votre consommation de cannabis ? | ☐ | ☐ | ☐ | ☐ | ☐ |
| 8. | Combien de fois avez-vous eu des problèmes dans le cadre de votre formation ou de votre travail à cause de votre consommation de cannabis ? | ☐ | ☐ | ☐ | ☐ | ☐ |

AU COURS DES 12 DERNIERS MOIS, vous est-il arrivé qu'un parent, un ami ou un médecin exprime des réserves sur votre consommation de cannabis ou vous conseille de réduire votre consommation ?

| | Non<br>*0* | Oui<br>*4* |
|---|---|---|
| 9. | ☐ | ☐ |

Laquelle de ces deux affirmations vous correspond-elle le mieux ?

| | « Je fume du cannabis par plaisir, parce que c'est quelque chose de spécial ».<br>*0* | « Je fume du cannabis par habitude, car cela fait partie de mon quotidien ».<br>*4* |
|---|---|---|
| 10. | ☐ | ☐ |

**German**

Wie häufig haben Sie in den LETZTEN 12 MONATEN Cannabis konsumiert?

| | 1 Mal pro Monat oder weniger<br>*0* | 2 bis 4 Mal pro Monat<br>*1* | 2 bis 3 Mal pro Woche<br>*2* | 4 bis 5 Mal pro Woche<br>*3* | Jeden Tag oder fast jeden Tag<br>*4* |
|---|---|---|---|---|---|
| 1. | ☐ | ☐ | ☐ | ☐ | ☐ |

Bitte denken Sie beim Beantworten der nächsten Fragen daran, wie es Ihnen in den LETZTEN 12 MONATEN ergangen ist:

| | *Kreuzen Sie in jeder Zeile ein Kästchen an.* | Nie<br>*0* | Weniger als 1-mal pro Monat<br>*1* | 1-mal pro Monat<br>*2* | 1-mal pro Woche<br>*3* | Täglich oder fast täglich<br>*4* |
|---|---|---|---|---|---|---|
| 2. | Wie häufig sind Sie mindestens 6 Stunden lang bekifft gewesen? | ☐ | ☐ | ☐ | ☐ | ☐ |
| 3. | Wie häufig haben Sie den Eindruck gehabt, mit dem Cannabisgebrauch nicht mehr aufhören zu können? | ☐ | ☐ | ☐ | ☐ | ☐ |
| 4. | Wie häufig sind Sie wegen des Cannabisgebrauches nicht fähig gewesen, das zu machen, was normalerweise von Ihnen erwartet wird? | ☐ | ☐ | ☐ | ☐ | ☐ |
| 5. | Wie häufig haben Sie morgens Cannabis genommen, um nach starkem Cannabisgebrauch am Vorabend wieder in Schwung zu kommen? | ☐ | ☐ | ☐ | ☐ | ☐ |
| 6. | Wie häufig haben Sie wegen Ihres Cannabisgebrauchs Probleme gehabt, sich zu erinnern oder zu konzentrieren? | ☐ | ☐ | ☐ | ☐ | ☐ |
| 7. | Wie häufig haben Sie wegen ihres Cannabisgebrauchs eine Freizeitaktivität aufgegeben, die Sie eigentlich machen wollten, zum Beispiel Ausgehen, Sport machen, oder Ihr Hobby treiben? | ☐ | ☐ | ☐ | ☐ | ☐ |
| 8. | Wie häufig hatten Sie während Ihrer Ausbildung oder ihrer Arbeit Probleme wegen Ihres Cannabiskonsums? | ☐ | ☐ | ☐ | ☐ | ☐ |

Hat in den LETZTEN 12 MONATEN ein Verwandter, ein Freund oder ein Arzt Bedenken wegen Ihres Cannabisgebrauchs geäussert oder Ihnen angeraten, den Gebrauch einzuschränken?

| | Nein<br>*0* | Ja<br>*4* |
|---|---|---|
| 9. | ☐ | ☐ |

Welche der beiden Aussagen trifft besser auf Sie zu?

| | „Ich rauche Cannabis zum Vergnügen, weil es etwas Besonderes ist."<br>*0* | „Ich rauche Cannabis aus Gewohnheit, weil es bei mir zum Alltag gehört."<br>*4* |
|---|---|---|
| 10. | ☐ | ☐ |

### *S1.9 Major depressive disorder (MDD)*

Symptoms of major depressive disorder were assessed using the Major Depression Inventory (WHO-MDI; Bech et al., 2001; Bech et al., 2015), developed to comprehensively cover the full spectrum of depressive symptoms as defined in DSM-IV major depression (APA, 1994) and ICD-10 mild, moderate, and severe depression (World Health Organization, 1992).

The WHO-MDI assesses "psychomotor changes" and "appetite changes" using two items each: item 8 (feeling restless) and item 9 (feeling subdued or slowed down) for psychomotor changes; item 11 (reduced appetite) and item 12 (increased appetite) for appetite changes. Only the higher score of each item pair (8 vs. 9 and 11 vs. 12) was retained (Bech et al., 2001; Olsen et al., 2003).

The French and German versions were translated by bilingual members of the research team using the forward-backward translation method.

**English**
OVER THE LAST TWO WEEKS, how often…

| | *Tick one box in each row.* | **All the time** *5* | **Most of the time** *4* | **Slightly more than half the time** *3* | **Slightly less than half the time** *2* | **Some of the time** *1* | **At no time** *0* |
|---|---|---|---|---|---|---|---|
| 1. | …have you felt low in spirits or sad? | ☐ | ☐ | ☐ | ☐ | ☐ | ☐ |
| 2. | …have you lost interest in your daily activities? | ☐ | ☐ | ☐ | ☐ | ☐ | ☐ |
| 3. | …have you felt lacking in energy and strength? | ☐ | ☐ | ☐ | ☐ | ☐ | ☐ |
| 4. | …have you felt less self-confident? | ☐ | ☐ | ☐ | ☐ | ☐ | ☐ |
| 5. | …have you had a bad conscience or feelings of guilt? | ☐ | ☐ | ☐ | ☐ | ☐ | ☐ |
| 6. | …have you felt that life wasn't worth living? | ☐ | ☐ | ☐ | ☐ | ☐ | ☐ |
| 7. | …have you had difficulty in concentrating, e.g. when reading the newspaper or watching television? | ☐ | ☐ | ☐ | ☐ | ☐ | ☐ |
| 8. | …have you felt very restless? | ☐ | ☐ | ☐ | ☐ | ☐ | ☐ |
| 9. | …have you felt subdued or slowed down? | ☐ | ☐ | ☐ | ☐ | ☐ | ☐ |
| 10. | …have you had trouble sleeping at night? | ☐ | ☐ | ☐ | ☐ | ☐ | ☐ |
| 11. | …have you suffered from reduced appetite? | ☐ | ☐ | ☐ | ☐ | ☐ | ☐ |
| 12. | …have you suffered from increased appetite? | ☐ | ☐ | ☐ | ☐ | ☐ | ☐ |

**French**

AU COURS DES DEUX DERNIÈRES SEMAINES, à quelle fréquence...

| *Cochez une case par ligne.* | | Tout le temps<br>*5* | La plupart du temps<br>*4* | Un peu plus de la moitié du temps<br>*3* | Un peu moins de la moitié du temps<br>*2* | Parfois<br>*1* | Jamais<br>*0* |
|---|---|---|---|---|---|---|---|
| 1. | …vous êtes-vous senti triste ou démoralisé ? | ☐ | ☐ | ☐ | ☐ | ☐ | ☐ |
| 2. | …avez-vous manqué d'intérêt pour vos activités quotidiennes ? | ☐ | ☐ | ☐ | ☐ | ☐ | ☐ |
| 3. | …avez-vous eu l'impression de manquer de force ou d'énergie ? | ☐ | ☐ | ☐ | ☐ | ☐ | ☐ |
| 4. | …vous êtes-vous senti moins sûr de vous ? | ☐ | ☐ | ☐ | ☐ | ☐ | ☐ |
| 5. | …avez-vous eu mauvaise conscience ou vous êtes-vous senti coupable ? | ☐ | ☐ | ☐ | ☐ | ☐ | ☐ |
| 6. | …avez-vous pensé que la vie ne valait pas la peine d'être vécue ? | ☐ | ☐ | ☐ | ☐ | ☐ | ☐ |
| 7. | …avez-vous eu du mal à vous concentrer, par exemple pour lire le journal ou regarder la télévision ? | ☐ | ☐ | ☐ | ☐ | ☐ | ☐ |
| 8. | …vous êtes-vous senti agité ? | ☐ | ☐ | ☐ | ☐ | ☐ | ☐ |
| 9. | …vous êtes-vous senti ramolli ou ralenti ? | ☐ | ☐ | ☐ | ☐ | ☐ | ☐ |
| 10. | …avez-vous eu du mal à dormir la nuit ? | ☐ | ☐ | ☐ | ☐ | ☐ | ☐ |
| 11. | …avez-vous eu moins d'appétit que d'habitude ? | ☐ | ☐ | ☐ | ☐ | ☐ | ☐ |
| 12. | …avez-vous eu plus d'appétit que d'habitude ? | ☐ | ☐ | ☐ | ☐ | ☐ | ☐ |

**German**

Bei den folgenden Fragen geht es darum, wie Sie sich IN DEN LETZTEN 2 WOCHEN gefühlt haben. Wieviel der Zeit...

| *Kreuzen Sie in jeder Zeile ein Kästchen an.* | | Ständig<br>*5* | Meistens<br>*4* | Etwas mehr als die Hälfte der Zeit<br>*3* | Etwas weniger als die Hälfte der Zeit<br>*2* | Ab und zu<br>*1* | Nie<br>*0* |
|---|---|---|---|---|---|---|---|
| 1. | ...haben Sie sich bedrückt oder traurig gefühlt? | ☐ | ☐ | ☐ | ☐ | ☐ | ☐ |
| 2. | ...hatten Sie kein Interesse an Ihren täglichen Aktivitäten? | ☐ | ☐ | ☐ | ☐ | ☐ | ☐ |
| 3. | ...hatten Sie das Gefühl, dass Ihnen Energie und Kräfte fehlen? | ☐ | ☐ | ☐ | ☐ | ☐ | ☐ |
| 4. | ...hatten Sie weniger Selbstvertrauen? | ☐ | ☐ | ☐ | ☐ | ☐ | ☐ |
| 5. | ...hatten Sie ein schlechtes Gewissen oder Schuldgefühle? | ☐ | ☐ | ☐ | ☐ | ☐ | ☐ |
| 6. | ...haben Sie gefühlt, dass das Leben nicht lebenswert ist? | ☐ | ☐ | ☐ | ☐ | ☐ | ☐ |
| 7. | ...fiel es Ihnen schwer, sich zu konzentrieren, z.B. beim Zeitunglesen oder Fernsehen? | ☐ | ☐ | ☐ | ☐ | ☐ | ☐ |
| 8. | ...fühlten Sie sich aussergewöhnlich rastlos? | ☐ | ☐ | ☐ | ☐ | ☐ | ☐ |
| 9. | ...fühlten Sie sich passiver als sonst? | ☐ | ☐ | ☐ | ☐ | ☐ | ☐ |
| 10. | ...hatten Sie Schwierigkeiten, nachts zu schlafen? | ☐ | ☐ | ☐ | ☐ | ☐ | ☐ |
| 11. | ...war Ihr Appetit schlechter als sonst? | ☐ | ☐ | ☐ | ☐ | ☐ | ☐ |
| 12. | ...war Ihr Appetit grösser als sonst? | ☐ | ☐ | ☐ | ☐ | ☐ | ☐ |

*S1.10 Attention deficit hyperactivity disorder (ADHD)*

Symptoms of attention-deficit/hyperactivity disorder (ADHD) were assessed using the six-question screener of the Adult ADHD Self-Report Scale (ASRS-v1.1; Kessler et al., 2005). The ASRS captures DSM-IV Criterion A symptoms of adult ADHD (APA, 1994).

The French and German versions were translated by bilingual members of the research team using the forward-backward translation method.

**English**
Think of how you have felt or behaved yourself IN THE PAST 12 MONTHS and tick the most relevant box in each row below.

| | *Tick one box in each row.* | **Never** *1* | **Rarely** *2* | **Sometimes** *3* | **Often** *4* | **Very often** *5* |
|---|---|---|---|---|---|---|
| 1. | How often do you have trouble wrapping up the final details of a project, once the challenging parts have been done? | ☐ | ☐ | ☐ | ☐ | ☐ |
| 2. | How often do you have difficulties getting things in order when you have to do a task that requires organization? | ☐ | ☐ | ☐ | ☐ | ☐ |
| 3. | How often do you have problems remembering appointments or obligations? | ☐ | ☐ | ☐ | ☐ | ☐ |
| 4. | When you are working on something that requires a lot of thinking, how often do you postpone or avoid the task? | ☐ | ☐ | ☐ | ☐ | ☐ |
| 5. | How often do you fidget or squirm with your hands or feet when you have to sit down for a long time? | ☐ | ☐ | ☐ | ☐ | ☐ |
| 6. | How often do you feel overly active and compelled to do things, like you were driven by a motor? | ☐ | ☐ | ☐ | ☐ | ☐ |

**French**
Pour chaque question, marquez d'une croix la réponse qui décrit le mieux comment vous vous êtes senti ou conduit AU COURS DES 12 DERNIERS MOIS.

| | *Cochez une case par ligne.* | **Jamais** *1* | **Rarement** *2* | **Quelque-fois** *3* | **Souvent** *4* | **Très souvent** *5* |
|---|---|---|---|---|---|---|
| 1. | A quelle fréquence avez-vous des difficultés à régler les derniers détails d'un projet une fois que le gros du travail a été fait ? | ☐ | ☐ | ☐ | ☐ | ☐ |
| 2. | A quelle fréquence avez-vous des difficultés à mettre les choses en ordre quand vous devez effectuer des travaux demandant de l'organisation ? | ☐ | ☐ | ☐ | ☐ | ☐ |
| 3. | A quelle fréquence avez-vous des problèmes à vous souvenir de vos rendez-vous ou obligations ? | ☐ | ☐ | ☐ | ☐ | ☐ |
| 4. | Lorsque vous devez effectuer un travail demandant beaucoup de réflexion, à quelle fréquence évitez-vous ou remettez-vous à plus tard le moment de vous mettre à la tâche ? | ☐ | ☐ | ☐ | ☐ | ☐ |
| 5. | A quelle fréquence vous tortillez-vous les mains ou devez-vous bouger les pieds si vous devez rester assis pour une longue période ? | ☐ | ☐ | ☐ | ☐ | ☐ |
| 6. | A quelle fréquence vous sentez-vous trop actif et poussé à faire les choses, comme si vous étiez poussé par un moteur ? | ☐ | ☐ | ☐ | ☐ | ☐ |

**German**
Markieren Sie bei den folgenden Fragen jeweils das Kästchen, das am besten beschreibt, wie Sie sich in den LETZTEN 12 MONATEN gefühlt und verhalten haben.

| | *Kreuzen Sie in jeder Zeile ein Kästchen an.* | **Nie** *1* | **Selten** *2* | **Manchmal** *3* | **Oft** *4* | **Sehr oft** *5* |
|---|---|---|---|---|---|---|
| 1. | Wie oft haben Sie Probleme, die letzten Feinheiten einer Arbeit zum Abschluss zu bringen, nachdem Sie die wesentlichen Punkte erledigt haben? | ☐ | ☐ | ☐ | ☐ | ☐ |
| 2. | Wie oft fällt es Ihnen schwer, Dinge auf die Reihe zu bekommen, wenn Sie an einer Aufgabe arbeiten, bei der Organisation gefragt ist? | ☐ | ☐ | ☐ | ☐ | ☐ |
| 3. | Wie oft haben Sie Probleme, sich an Termine oder Verabredungen zu erinnern? | ☐ | ☐ | ☐ | ☐ | ☐ |
| 4. | Wie oft vermeiden oder verzögern Sie, eine Aufgabe zu beginnen, wenn es sich um eine Aufgabe handelt, bei der sehr viel Nachdenken gefragt ist? | ☐ | ☐ | ☐ | ☐ | ☐ |
| 5. | Wie oft sind Ihre Hände bzw. Füße bei langem Sitzen ständig in Bewegung („rumzappeln“)? | ☐ | ☐ | ☐ | ☐ | ☐ |
| 6. | Wie oft fühlen Sie sich übermäßig aktiv und verspüren den Drang Dinge zu tun, als ob Sie von einem Motor angetrieben würden? | ☐ | ☐ | ☐ | ☐ | ☐ |

### *S1.11 Borderline pers. disorder (BPD)*

Symptoms of borderline personality disorder were assessed using the McLean Screening Instrument for Borderline Personality Disorder (MSI-BPD; Melartin et al., 2009; Zanarini et al., 2003). The MSI-BPD is based on DSM-IV diagnostic criteria (APA, 1994).

The French version was translated by bilingual members of the research team using the forward-backward translation method.

**English**

Please read attentively the questions below and decide if they correspond to you or not by checking the box "true" or "false", even if you are not completely sure of your answer.

| | *Tick one box in each row* | **Yes** *1* | **No** *0* |
|---|---|---|---|
| 1. | Have any of your closest relationships been troubled by a lot of arguments or repeated breakups? | ☐ | ☐ |
| 2. | Have you deliberately hurt yourself physically (e.g. punched yourself, cut yourself, burned yourself)? | ☐ | ☐ |
| 3. | Have you had at least two othe problems with impulsivity (e.g. eating binges and spending sprees, drinking too much and verbal outburst)? | ☐ | ☐ |
| 4. | Have you been extremely moody? | ☐ | ☐ |
| 5. | Have you felt very angry a lot of the time? How about often acted in an angry or sarcastic manner? | ☐ | ☐ |
| 6. | Have you often been distrustful of the other people? | ☐ | ☐ |
| 7. | Have you frequently felt unreal or as if things around you were unreal? | ☐ | ☐ |
| 8. | Have you chronically felt empty? | ☐ | ☐ |
| 9. | Have you often felt that you had no idea of who you are or that you have no identity? | ☐ | ☐ |
| 10. | Have you made desperate efforts to avoid feeling abandoned or being abandoned (e.g. repeatedly called someone to reassure yourself that he or she still cared, begged them not to leave you, clung to them physically)? | ☐ | ☐ |

**French**

Ci-dessous une série de questions. Veuillez lire attentivement chaque question et décider si elle vous correspond ou non en cochant la case « vrai » ou « faux », même si vous n'êtes pas tout à fait sûr de votre réponse

| | *Cochez une case par ligne.* | **Vrai** *1* | **Faux** *0* |
|---|---|---|---|
| 1. | Est-ce que votre relation avec une personne très proche a été perturbée par de nombreuses disputes ou ruptures répétées ? | ☐ | ☐ |
| 2. | Vous êtes vous délibérément blessé physiquement (par ex. vous frapper, couper, brûler) ? | ☐ | ☐ |
| 3. | Avez-vous eu au moins deux autres problèmes d'impulsivité (par ex. manger à l'excès et faire des dépenses inutiles, boire et exploser verbalement) ? | ☐ | ☐ |
| 4. | Avez-vous été d'humeur extrêmement changeante ? | ☐ | ☐ |
| 5. | Vous êtes-vous souvent senti très en colère ? Avez-vous souvent agi sous l'effet de la colère ou avec sarcasme ? | ☐ | ☐ |
| 6. | Avez-vous souvent été méfiant envers d'autres personnes ? | ☐ | ☐ |
| 7. | Vous êtes-vous fréquemment senti irréel ou comme si les choses autour de vous étaient irréelles ? | ☐ | ☐ |
| 8. | Vous êtes-vous senti chroniquement vide ? | ☐ | ☐ |
| 9. | Avez-vous souvent ressenti que vous n'aviez aucune idée de qui vous étiez ou que vous n'aviez pas d'identité ? | ☐ | ☐ |
| 10. | Avez-vous fait des efforts désespérés pour éviter de vous sentir ou d'être abandonné (par ex. avoir appelé quelqu'un à plusieurs reprises pour se rassurer qu'il ou elle se souciait encore de vous, le ou la supplier de ne pas vous quitter, se cramponner à lui ou elle) ? | ☐ | ☐ |

**German**

Im Folgenden eine Reihe von Fragen. Lesen Sie diese bitte aufmerksam und entscheiden Sie, was AM EHESTEN auf Sie zutrifft, auch wenn Sie sich nicht ganz sicher sind. Ist es also eher „richtig“ oder eher „falsch“?

| | *Kreuzen Sie ein Kästchen pro Zeile an.* | **Richtig** *1* | **Falsch** *0* |
|---|---|---|---|
| 1. | Ist eine Ihrer engsten Beziehungen häufig durch Streitigkeiten oder wiederholte Trennungen belastet worden? | ☐ | ☐ |
| 2. | Haben Sie sich absichtlich selbst körperlich verletzt (z.B. sich selbst geschlagen, geschnitten, verbrannt)? | ☐ | ☐ |
| 3. | Hatten Sie wenigstens zwei andere Probleme mit Ihrer Impulsivität (z.B. Fressorgien, Kaufräusche, zu viel getrunken, verbale Zorn- oder Wutausbrüche)? | ☐ | ☐ |
| 4. | Sind Sie extrem launisch gewesen? | ☐ | ☐ |
| 5. | Waren Sie häufig wütend? Haben Sie häufig zornig oder sarkastisch reagiert? | ☐ | ☐ |
| 6. | Waren Sie anderen Leuten häufig misstrauisch gegenüber? | ☐ | ☐ |
| 7. | Haben Sie sich häufig unwirklich gefühlt oder dachten Sie, die Dinge um sie herum wären unwirklich? | ☐ | ☐ |
| 8. | Haben Sie sich chronisch leer gefühlt? | ☐ | ☐ |
| 9. | Hatten Sie häufig das Gefühl, dass Sie nicht wussten, wer Sie sind oder dass Sie keine eigene Identität hätten? | ☐ | ☐ |
| 10. | Haben sie verzweifelt versucht, Gefühle des Verlassenseins zu vermeiden? Oder fühlten Sie sich verlassen? (Haben Sie z.B. wiederholt jemanden angerufen, um sich zu versichern, dass sich diese Person um Sie sorgt? Oder haben Sie darum gebettelt, dass Sie nicht verlassen werden, oder hingen Sie wie eine Klette an dieser Person?) | ☐ | ☐ |

### *S1.12 Social anxiety disorder (SAD)*

Symptoms of social anxiety disorder were assessed using the Clinically Useful Social Anxiety Disorder Outcome Scale (CUSADOS; Dalrymple et al., 2013). The CUSADOS was developed based on content from diagnostic interviews, including the Structured Clinical Interview for DSM-IV (SCID; First et al., 1997) and the Psychiatric Diagnostic Screening Questionnaire (Zimmerman & Mattia, 2001).

The French and German versions were translated by bilingual members of the research team using the forward-backward translation method.

**English**
How well the item describes you DURING THE PAST WEEK, including today?

| | *Tick one box in each row.* | **Not at all true** *0* | **Rarely true** *1* | **Sometimes true** *2* | **Often true** *3* | **Almost always true** *4* |
|---|---|---|---|---|---|---|
| 1. | I was very afraid of being judged by others | ☐ | ☐ | ☐ | ☐ | ☐ |
| 2. | I was extremely afraid of social situations | ☐ | ☐ | ☐ | ☐ | ☐ |
| 3. | I was worried that I would make a mistake in front of others and look foolish | ☐ | ☐ | ☐ | ☐ | ☐ |
| 4. | I avoided social situations where people might pay attention to me | ☐ | ☐ | ☐ | ☐ | ☐ |
| 5. | I was afraid to walk into a crowded room because everyone would look at me | ☐ | ☐ | ☐ | ☐ | ☐ |
| 6. | I was afraid of eating, drinking, or writing in front of other people | ☐ | ☐ | ☐ | ☐ | ☐ |
| 7. | I was very concerned that people would notice that I was anxious | ☐ | ☐ | ☐ | ☐ | ☐ |
| 8. | I avoided eating, drinking, or writing in front of people | ☐ | ☐ | ☐ | ☐ | ☐ |
| 9. | I worried that I would say something stupid in front of other people | ☐ | ☐ | ☐ | ☐ | ☐ |
| 10. | I was worried about being criticized by other people | ☐ | ☐ | ☐ | ☐ | ☐ |
| 11. | I was worried that other people may not like me | ☐ | ☐ | ☐ | ☐ | ☐ |
| 12. | After I was criticized, I thought about it for a long time | ☐ | ☐ | ☐ | ☐ | ☐ |

**French**
A quel point les propositions ci-dessous décrivent votre état DURANT LA SEMAINE ÉCOULÉE, y compris aujourd'hui ?

| | *Cochez une case par ligne.* | **Presque jamais vrai** *0* | **Rarement vrai** *1* | **Parfois vrai** *2* | **Souvent vrai** *3* | **Presque toujours vrai** *4* |
|---|---|---|---|---|---|---|
| 1. | J'ai eu très peur d'être jugé par les autres | ☐ | ☐ | ☐ | ☐ | ☐ |
| 2. | J'ai eu extrêmement peur des situations sociales | ☐ | ☐ | ☐ | ☐ | ☐ |
| 3. | J'ai craint de faire une erreur devant les autres et d'avoir l'air bête | ☐ | ☐ | ☐ | ☐ | ☐ |
| 4. | J'ai évité les situations sociales qui auraient pu attirer l'attention des gens sur moi | ☐ | ☐ | ☐ | ☐ | ☐ |
| 5. | J'ai eu peur d'entrer dans une salle bondée parce que tout le monde m'aurait regardé | ☐ | ☐ | ☐ | ☐ | ☐ |
| 6. | J'ai eu peur de manger, boire ou écrire devant d'autres personnes | ☐ | ☐ | ☐ | ☐ | ☐ |
| 7. | J'ai été préoccupé du fait que les gens remarquent que j'étais anxieux | ☐ | ☐ | ☐ | ☐ | ☐ |
| 8. | J'ai évité de manger, boire, ou écrire devant des personnes | ☐ | ☐ | ☐ | ☐ | ☐ |
| 9. | J'ai eu peur de dire quelque chose de stupide devant d'autres personnes | ☐ | ☐ | ☐ | ☐ | ☐ |
| 10. | J'ai eu peur d'être critiqué par d'autres personnes | ☐ | ☐ | ☐ | ☐ | ☐ |
| 11. | J'ai eu peur que les autres puissent ne pas m'apprécier | ☐ | ☐ | ☐ | ☐ | ☐ |
| 12. | Après avoir été critiqué, j'y ai pensé pendant longtemps | ☐ | ☐ | ☐ | ☐ | ☐ |

**German**

Bis zu welchem Punkt beschreiben die folgenden Aussagen Ihren Gefühlszustand der LETZTEN WOCHE, heute eingeschlossen?

| | *Kreuzen Sie ein Kästchen pro Zeile an.* | **Fast nie wahr** *0* | **Selten wahr** *1* | **Manchmal wahr** *2* | **Oft wahr** *3* | **Fast immer wahr** *4* |
|---|---|---|---|---|---|---|
| 1. | Ich hatte grosse Angst davor, von anderen beurteilt zu werden. | ☐ | ☐ | ☐ | ☐ | ☐ |
| 2. | Ich hatte totale Angst vor Situationen mit vielen Menschen. | ☐ | ☐ | ☐ | ☐ | ☐ |
| 3. | Ich war besorgt, ich könnte einen Fehler vor anderen machen und dumm aussehen. | ☐ | ☐ | ☐ | ☐ | ☐ |
| 4. | Ich vermied gesellschaftliche Situationen, in denen Leute mir Aufmerksamkeit schenken könnten. | ☐ | ☐ | ☐ | ☐ | ☐ |
| 5. | Ich hatte Angst davor, in einen überfüllten Raum zu gehen, weil mich jeder anstarren würde. | ☐ | ☐ | ☐ | ☐ | ☐ |
| 6. | Ich hatte Angst, in Anwesenheit von anderen zu essen, zu trinken oder zu schreiben. | ☐ | ☐ | ☐ | ☐ | ☐ |
| 7. | Ich befürchtete, andere Personen könnten merken, dass ich ängstlich war. | ☐ | ☐ | ☐ | ☐ | ☐ |
| 8. | Ich vermied es, in Anwesenheit von anderen Personen zu essen, zu trinken oder zu schreiben. | ☐ | ☐ | ☐ | ☐ | ☐ |
| 9. | Ich hatte Angst, etwas Dummes vor anderen Leuten zu sagen. | ☐ | ☐ | ☐ | ☐ | ☐ |
| 10. | Ich war besorgt, von anderen kritisiert zu werden. | ☐ | ☐ | ☐ | ☐ | ☐ |
| 11. | Ich war besorgt, dass andere Leute mich nicht leiden können. | ☐ | ☐ | ☐ | ☐ | ☐ |
| 12. | Nach einer Kritik musste ich noch eine lange Zeit darüber nachdenken. | ☐ | ☐ | ☐ | ☐ | ☐ |

## Supplementary Material S2: Sample characteristics

Table S2.1: Proportion of participants screened positive for behavioral addictions, substance use disorders, and mental health problems at baseline and follow-up, according to the respective instrument cut-offs

| | Psychometric scales and cut-off criteria | Time frame | n | | Screened positive (%) | |
|---|---|---|---|---|---|---|
| | | | bl | fu | bl | fu |
| **Behavioral addictions** | | | | | | |
| Gambling | Participants were screened positive for mild **gambling disorder** (APA, 2013) using a polythetic cut-off, defined as meeting ≥ 4 out of 9 criteria from the DSM-IV Pathological Gambling Diagnostic Form (Office of Alcoholism and Substance Abuse Services, 2011). | 12 M | 5505 | 5090 | 1.34 | 0.75 |
| Gaming | Participants were screened positive for **problematic gaming** using a polythetic cut-off, defined as meeting ≥ 4 out of 7 criteria from the 7-item Game Addiction Scale (Lemmens et al., 2009). A criterion was coded as met if the item was endorsed with a score of at least 3 ('sometimes') on a 5-point Likert-type scale ranging from 1 ('never') to 5 ('very often'). | 6 M | 5511 | 5091 | 7.00 | 4.46 |
| Internet | Participants were screened positive for **problematic internet use** using a cut-off score of ≥ 28 out of 56 points on the Compulsive Internet Use Scale (CIUS; Meerkerk et al., 2010; Meerkerk et al., 2009). | – | 5512 | 5089 | 4.74 | 5.01 |
| Pornography | Participants were screened positive for **problematic internet pornography use** using a polythetic cut-off, defined as meeting ≥ 3 out of 6 criteria from the Online Sexual Compulsivity subscale of the Internet Sex Screening Test (ISST; Carnes et al., 2009; Delmonico & Miller, 2003). | 12 M | 5427 | 5090 | 7.04 | 7.37 |
| Smartphone | Participants were screened positive for **problematic smartphone use** using a cut-off score of ≥ 31 out of 50 points on the Smartphone Addiction Scale – Short Version (SAS-SV; Haug et al., 2015; Kwon, Kim, et al., 2013). | – | 5513 | 5089 | 8.14 | 10.53 |
| Work | Participants were screened positive for **work addiction** using a polythetic cut-off, defined as meeting ≥ 4 out of 7 criteria from the Bergen Work Addiction Scale (BWAS; Andreassen et al., 2012). A criterion was coded as met if the item was endorsed with a score of at least 3 ('often') on a 5-point Likert-type scale ranging from 1 ('never') to 5 ('always'). | 12 M | 5434 | 5052 | 8.06 | 7.62 |
| **Substance use disorders** | | | | | | |
| Alcohol use disorder (AUD) | Participants were screened positive for moderate **alcohol use disorder** using a polythetic cut-off, defined as meeting ≥ 4 out of 11 criteria based on the DSM-5 (APA, 2013), assessed with items from Knight et al. (2002) and Grant et al. (2003). | 12 M | 5507 | 5087 | 8.84 | 7.16 |
| Cannabis use disorder (CUD) | Participants were screened positive for **cannabis use disorder** using a cut-off score of ≥ 8 out of 40 points on the Cannabis Use Disorder Identification Test – Revised (CUDIT-R; Annaheim et al., 2010), a revised version of the original scale by Adamson and Sellman (2003). | 12 M | 5502 | 5090 | 8.00 | 6.68 |
| **Other mental health problems** | | | | | | |
| Major depressive disorder (MDD) | Participants were screened positive for **major depressive disorder** using a cut-off score of ≥ 21 out of 50 points on the Major Depression Inventory (WHO-MDI; Bech et al., 2001; Bech et al., 2015; Olsen et al., 2003). | 2 W | 5435 | 5054 | 7.89 | 9.08 |
| Attention deficit hyperactivity disorder (ADHD) | Participants were screened positive for **attention-deficit/hyperactivity disorder** (ADHD) using a polythetic cut-off, defined as meeting ≥ 4 out of 6 criteria from the Adult ADHD Self-Report Scale (ASRS-v1.1; Kessler et al., 2005). The first three criteria were coded as met if the item was endorsed with a score of at least 3 ('sometimes'), while the last three required a score of at least 4 ('often') on a 5-point Likert-type scale ranging from 1 ('never') to 5 ('very often'). | 12 M | 5509 | 5088 | 7.75 | 9.43 |
| Borderline pers. disorder (BPD) | Participants were screened positive for **borderline personality disorder** using a polythetic cut-off, defined as meeting ≥ 7 out of 10 criteria from the McLean Screening Instrument for Borderline Personality Disorder (Melartin et al., 2009; Zanarini et al., 2003). | LT | 5426 | 5054 | 4.77 | 4.12 |
| Social anxiety disorder (SAD) | Participants were screened positive for **social anxiety disorder** using a cut-off score of ≥ 16 out of 48 points on the Clinically Useful Social Anxiety Disorder Outcome Scale (CUSADOS; Dalrymple et al., 2013). | 1 W | 5425 | 5053 | 16.83 | 14.55 |

Note: The cut-off criteria and prevalence rates reported in this table are used solely for sample description. In all further analyses, BAs, SUDs, and MHPs were conceptualized as continuous latent variables indicating symptom severity. Time frame = denoted as reference period in number of months (M), weeks (W), or not explicitly specified (−); n = participants who completed the respective scale; for correlations, bl = baseline, fu = follow-up.

## Supplementary Material S3: Main analysis

Table S3.1: Main analysis: Fit indices of latent change score models.

| | BEHAVIORAL ADDICITON (BA) | | | | | | | | | | | | | | | | | |
|---|---|---|---|---|---|---|---|---|---|---|---|---|---|---|---|---|---|---|
| | **Gambling** | | | **Gaming** | | | **Internet** | | | **Pornography** | | | **Smartphone** | | | **Work** | | |
| | ***RMSEA [95%CI]*** | ***CFI*** | ***TLI*** | ***RMSEA [95%CI]*** | ***CFI*** | ***TLI*** | ***RMSEA [95%CI]*** | ***CFI*** | ***TLI*** | ***RMSEA [95%CI]*** | ***CFI*** | ***TLI*** | ***RMSEA [95%CI]*** | ***CFI*** | ***TLI*** | ***RMSEA [95%CI]*** | ***CFI*** | ***TLI*** |
| **Behavioral addictions** | | | | | | | | | | | | | | | | | | |
| Gambling | **.019** [.018, .020] | **.980** | **.979** | | | | | | | | | | | | | | | |
| Gaming | **.033** [.032, .033] | **.957** | **.957** | **.053** [.052, .054] | *.922* | *.922* | | | | | | | | | | | | |
| Internet | **.012** [.011, .014] | **.980** | **.978** | **.031** [.030, .032] | **.975** | **.972** | **.046** [.046, .047] | *.943* | *.942* | | | | | | | | | |
| Pornography | **.028** [.027, .029] | **.962** | **.962** | **.045** [.044, .046] | *.948* | *.948* | **.051** [.050, .052] | *.919* | *.921* | **.042** [.041, .043] | **.952** | **.952** | | | | | | |
| Smartphone | **.021** [.020, .023] | **.963** | **.961** | **.044** [.043, .045] | **.951** | *.947* | **.051** [.050, .052] | *.929* | *.929* | **.038** [.037, .039] | *.943* | *.937* | **.047** [.046, .048] | *.940* | *.939* | | | |
| **Substance use disorders** | | | | | | | | | | | | | | | | | | |
| Alcohol use disorder | **.013** [.012, .014] | **.965** | **.963** | **.024** [.023, .025] | **.970** | **.968** | **.035** [.034, .035] | **.953** | **.952** | **.021** [.020, .022] | **.951** | *.948* | **.031** [.030, .032] | **.958** | **.957** | **.029** [.028, .030] | *.938* | *.934* |
| Cannabis use disorder | **.011** [.009, .012] | **.996** | **.996** | **.022** [.021, .023] | **.989** | **.988** | **.035** [.035, .036] | **.963** | **.964** | **.018** [.017, .019] | **.994** | **.993** | **.032** [.031, .032] | **.975** | **.975** | **.029** [.028, .030] | **.983** | **.982** |
| **Mental health problems** | | | | | | | | | | | | | | | | | | |
| Major depression | **.017** [.016, .018] | **.985** | **.985** | **.035** [.035, .036] | **.968** | **.968** | **.044** [.043, .044] | *.942* | *.943* | **.027** [.026, .028] | **.980** | **.980** | **.040** [.039, .041] | **.953** | **.955** | **.049** [.048, .050] | *.938* | *.938* |
| ADHD | **.025** [.024, .026] | **.956** | **.952** | **.047** [.045, .048] | **.954** | *.949* | **.053** [.053, .054] | *.928* | *.928* | **.041** [.039, .042] | *.947* | *.941* | **.052** [.051, .053] | *.934* | *.934* | **.054** [.052, .055] | *.922* | *.914* |
| Borderline personality disorder | **.014** [.013, .015] | **.977** | **.975** | **.028** [.028, .029] | **.970** | **.968** | **.039** [.039, .040] | *.948* | *.948* | **.023** [.022, .024] | **.969** | **.967** | **.034** [.034, .035] | **.957** | **.957** | **.038** [.037, .038] | *.935* | *.931* |
| Social anxiety | **.029** [.028, .030] | **.976** | **.975** | **.044** [.043, .044] | **.960** | **.960** | **.043** [.043, .044] | *.945* | *.946* | **.040** [.039, .040] | **.970** | **.970** | **.043** [.043, .044] | **.951** | **.952** | **.049** [.048, .050] | **.951** | **.951** |

Note: BAs, SUDs, and MHPs were modeled as continuous latent variables indicating symptom severity. RMSEA = root mean square error of approximation; CFI = comparative fit index; TLI = Tucker–Lewis index; gambling = gambling disorder; gaming = problematic gaming; internet = problematic internet use; pornography = problematic pornography use; smartphone = problematic smartphone use; work = work addiction; ADHD = attention-deficit/hyperactivity disorder. **Bold** font is used to indicate good fit; *italic* font is used to indicate acceptable fit.

**Supplementary Material S4: Sensitivity analysis**

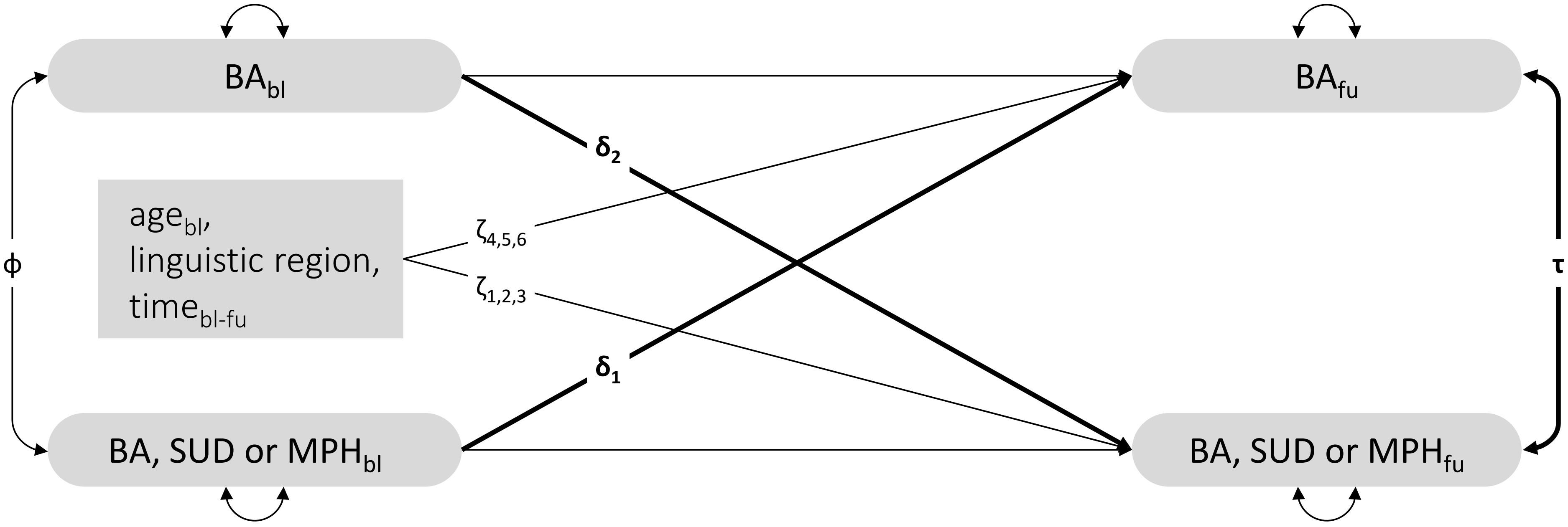


Figure S3.1: Sensitivity analysis: Cross-lagged panel model estimates of correlated change (τ) and directional change (δ1, δ2) between behavioral addictions (BA) and one other BA, substance use disorder (SUD), or mental health problem (MHP).

Remarks: The model was estimated separately for each behavioral addiction (gambling, gaming, internet, pornography, smartphone, and work) in combination with one other BA, a SUD (alcohol use disorder, cannabis use disorder), or an MHP (major depressive disorder, ADHD, borderline personality disorder, and social anxiety disorder). Rounded grey shapes depict continuous, latent variables for symptoms of mental health problems. The grey rectangle represents observed variables (age$_{bl}$, linguistic region, time$_{bl-fu}$). Residuals and variances are illustrated as double-headed arrows entering a grey shape. Double-headed arrows (τ, Φ) represent correlations between two grey shapes, while single-headed arrows indicate paths (δ, ζ). Covariances between age, language, duration, BA$_{bl}$, and BA/SUD/MHP$_{bl}$, as well as means, are estimated but not shown, for visual clarity.

Table S3.1: Sensitivity analysis: Fit indices of cross-lagged panel models.

| | BEHAVIORAL ADDICITON (BA) | | | | | | | | | | | | | | | | | |
|---|---|---|---|---|---|---|---|---|---|---|---|---|---|---|---|---|---|---|
| | **Gambling** | | | **Gaming** | | | **Internet** | | | **Pornography** | | | **Smartphone** | | | **Work** | | |
| | ***RMSEA [95%CI]*** | ***CFI*** | ***TLI*** | ***RMSEA [95%CI]*** | ***CFI*** | ***TLI*** | ***RMSEA [95%CI]*** | ***CFI*** | ***TLI*** | ***RMSEA [95%CI]*** | ***CFI*** | ***TLI*** | ***RMSEA [95%CI]*** | ***CFI*** | ***TLI*** | ***RMSEA [95%CI]*** | ***CFI*** | ***TLI*** |
| **Behavioral addictions** | | | | | | | | | | | | | | | | | | |
| Gambling | **.019** [.018; .020] | **.981** | **.979** | | | | | | | | | | | | | | | |
| Gaming | **.033** [.032; .033] | **.957** | **.957** | **.053** [.052; .054] | *.923* | *.923* | | | | | | | | | | | | |
| Internet | **.012** [.011; .014] | **.980** | **.979** | **.031** [.030; .032] | **.975** | **.972** | **.046** [.045; .047] | *.943* | *.943* | | | | | | | | | |
| Pornography | **.028** [.027; .029] | **.962** | **.962** | **.045** [.044; .045] | *.948* | *.948* | **.051** [.050; .052] | *.919* | *.921* | **.042** [.041; .043] | **.952** | **.953** | | | | | | |
| Smartphone | **.021** [.020; .022] | **.963** | **.961** | **.044** [.043; .045] | **.951** | *.947* | **.051** [.050; .051] | *.929* | *.929* | **.038** [.037; .039] | *.943* | *.938* | **.047** [.046; .048] | *.940* | *.940* | | | |
| **Substance use disorders** | | | | | | | | | | | | | | | | | | |
| Alcohol use disorder | **.013** [.012; .014] | **.965** | **.964** | **.024** [.023; .025] | **.970** | **.968** | **.035** [.034; .035] | **.953** | **.952** | **.021** [.020; .022] | **.951** | **.948** | **.031** [.030; .032] | **.958** | **.958** | **.029** [.028; .030] | *.938* | *.934* |
| Cannabis use disorder | **.010** [.009; .012] | **.996** | **.996** | **.022** [.021; .023] | **.989** | **.988** | **.035** [.035; .036] | **.963** | **.964** | **.018** [.017; .019] | **.994** | **.993** | **.032** [.031; .032] | **.975** | **.975** | **.029** [.028; .030] | **.983** | **.983** |
| **Mental health problems** | | | | | | | | | | | | | | | | | | |
| Major depression | **.017** [.016; .018] | **.985** | **.985** | **.035** [.034; .036] | **.968** | **.968** | **.043** [.043; .044] | *.942* | *.943* | **.027** [.026; .028] | **.980** | **.980** | **.040** [.039; .041] | **.953** | **.955** | **.049** [.048; .049] | **.938** | **.938** |
| ADHD | **.025** [.023; .026] | **.956** | **.953** | **.046** [.045; .047] | **.954** | *.950* | **.053** [.052; .054] | *.928* | *.928* | **.041** [.039; .042] | *.947* | *.941* | **.052** [.051; .053] | *.934* | *.935* | **.053** [.052; .055] | *.922* | *.914* |
| Borderline personality disorder | **.014** [.013; .015] | **.977** | **.976** | **.028** [.027; .029] | **.970** | **.968** | **.039** [.038; .040] | *.948* | *.948* | **.023** [.022; .024] | **.969** | **.967** | **.034** [.034; .035] | **.957** | **.957** | **.037** [.037; .038] | *.935* | *.931* |
| Social anxiety | **.029** [.028; .029] | **.976** | **.976** | **.043** [.043; .044] | **.960** | **.960** | **.043** [.042; .044] | *.946* | *.947* | **.040** [.039; .040] | **.970** | **.970** | **.043** [.043; .044] | **.951** | **.953** | **.049** [.048; .049] | **.951** | **.951** |

Note: BAs, SUDs, and MHPs were modeled as continuous latent variables indicating symptom severity. RMSEA = root mean square error of approximation; CFI = comparative fit index; TLI = Tucker–Lewis index; gambling = gambling disorder; gaming = problematic gaming; internet = problematic internet use; pornography = problematic internet pornography use; smartphone = problematic smartphone use; work = work addiction; ADHD = attention-deficit/hyperactivity disorder. **Bold** font is used to indicate good fit; *italic* font is used to indicate acceptable fit.

Table S3.2: Sensitivity analysis: Correlated change (τ) between symptoms of behavioral addictions and mental health problems in the cross-lagged panel model.

| | BEHAVIORAL ADDICITON (BA) | | | | | | | | | | | | | | | | | |
|---|---|---|---|---|---|---|---|---|---|---|---|---|---|---|---|---|---|---|
| | Gambling | | | Gaming | | | Internet | | | Pornography | | | Smartphone | | | Work | | |
| | *r* | *95%CI* | *p* | *r* | *95%CI* | *p* | *r* | *95%CI* | *p* | *r* | *95%CI* | *p* | *r* | *95%CI* | *p* | *r* | *95%CI* | *p* |
| **Behavioral addictions** | | | | | | | | | | | | | | | | | | |
| Gaming | **.167** | [.003, .331] | .045 [S] | | | | | | | | | | | | | | | |
| Internet | **.209** | [.060, .358] | .006 [S] | **.234** | [.184, .283] | <.001 [S] | | | | | | | | | | | | |
| Pornography | **.313** | [.011, .616] | .042 [M] | **.136** | [.064, .208] | <.001 [S] | **.321** | [.267, .376] | <.001 [M] | | | | | | | | | |
| Smartphone | **.283** | [.136, .429] | <.001 [S] | **.191** | [.141, .240] | <.001 [S] | **.572** | [.545, .599] | <.001 [L] | **.286** | [.229, .343] | <.001 [S] | | | | | | |
| Work | **.147** | [.019, .274] | .024 [S] | **.052** | [.007, .096] | .022 | **.134** | [.097, .171] | <.001 [S] | **.157** | [.097, .217] | <.001 [S] | **.119** | [.081, .157] | <.001 [S] | | | |
| **Substance use disorders** | | | | | | | | | | | | | | | | | | |
| AUD | .169 | [-.060, .397] | .148 [S] | **.146** | [.071, .221] | <.001 [S] | **.182** | [.120, .243] | <.001 [S] | **.253** | [.159, .347] | <.001 [S] | **.211** | [.151, .271] | <.001 [S] | **.127** | [.060, .193] | <.001 [S] |
| CUD | .064 | [-.195, .322] | .630 | **.153** | [.061, .244] | .001 [S] | .019 | [-.061, .098] | .643 | .122 | [-.011, .254] | .073 [S] | .008 | [-.077, .092] | .862 | **.094** | [.011, .178] | .027 |
| **Mental health problems** | | | | | | | | | | | | | | | | | | |
| MDD | **.247** | [.124, .371] | <.001 [S] | **.238** | [.195, .280] | <.001 [S] | **.228** | [.192, .264] | <.001 [S] | **.183** | [.118, .247] | <.001 [S] | **.179** | [.140, .217] | <.001 [S] | **.392** | [.360, .425] | <.001 [M] |
| ADHD | **.255** | [.101, .408] | .001 [S] | **.213** | [.164, .262] | <.001 [S] | **.277** | [.240, .313] | <.001 [S] | **.215** | [.153, .276] | <.001 [S] | **.247** | [.209, .286] | <.001 [S] | **.226** | [.190, .262] | <.001 [S] |
| BPD | **.348** | [.185, .510] | <.001 [M] | **.217** | [.161, .274] | <.001 [S] | **.212** | [.164, .261] | <.001 [S] | **.267** | [.191, .343] | <.001 [S] | **.211** | [.161, .261] | <.001 [S] | **.264** | [.217, .310] | <.001 [S] |
| SAD | **.266** | [.123, .409] | <.001 [S] | **.211** | [.166, .256] | <.001 [S] | **.238** | [.203, .274] | <.001 [S] | **.242** | [.182, .302] | <.001 [S] | **.232** | [.193, .270] | <.001 [S] | **.248** | [.212, .284] | <.001 [S] |

Note: BAs, SUDs, and MHPs were modeled as continuous latent variables indicating symptom severity. Gambling = gambling disorder; gaming = problematic gaming; internet = problematic internet use; pornography = problematic internet pornography use; smartphone = problematic smartphone use; work = work addiction; AUD = alcohol use disorder; CUD = cannabis use disorder; MDD = major depressive disorder; ADHD = attention-deficit/hyperactivity disorder; BPD = borderline personality disorder; SAD = social anxiety disorder; r = Pearson correlation coefficient; CI = confidence interval; ES = effect size according to Peterson and Brown (2005).

Full Information Maximum Likelihood (FIML) was used, allowing for the inclusion of participants with missing values under the 'missing at random' assumption (n = 5611). **Bold** font indicates significant coefficients ($p < .05$). [S/M/L] = small/medium/large effect size according to Cohen (1988).

Table S3.3: Sensitivity analysis: Directional change (δ1 and δ2) between symptoms of behavioral addictions and mental health problems in the cross-lagged panel model, standardized path coefficients.

| | | BEHAVIORAL ADDICITON (BA) | | | | | | | | | | | | | | | | | |
|---|---|---|---|---|---|---|---|---|---|---|---|---|---|---|---|---|---|---|---|
| Pathway | | Gambling | | | Gaming | | | Internet | | | Pornography | | | Smartphone | | | Work | | |
| | | *β* | *95%CI* | *p* | *β* | *95%CI* | *p* | *β* | *95%CI* | *p* | *β* | *95%CI* | *p* | *β* | *95%CI* | *p* | *β* | *95%CI* | *p* |
| **Behavioral addictions** | | | | | | | | | | | | | | | | | | | |
| Gaming | $\delta_1$ Gaming bl → BA fu | **.117** | [.017, .218] | .022$^{M}$ | | | | | | | | | | | | | | | |
| | $\delta_2$ BA bl → Gaming fu | .011 | [-.089, .111] | .829 | | | | | | | | | | | | | | | |
| Internet | $\delta_1$ Internet bl → BA fu | **.096** | [.006, .186] | .036$^{M}$ | **.058** | [.021, .094] | .002$^{S}$ | | | | | | | | | | | | |
| | $\delta_2$ BA bl → Internet fu | .027 | [-.057, .110] | .531 | **-.076** | [-.114, -.038] | <.001$^{M}$ | | | | | | | | | | | | |
| Pornography | $\delta_1$ Pornography bl → BA fu | .041 | [-.066, .148] | .455$^{S}$ | **.052** | [.007, .097] | .024$^{S}$ | **.134** | [.092, .175] | <.001$^{L}$ | | | | | | | | | |
| | $\delta_2$ BA bl → Pornography fu | -.061 | [-.187, .065] | .343$^{S}$ | .038 | [-.005, .081] | .085$^{S}$ | **.099** | [.054, .144] | <.001$^{M}$ | | | | | | | | | |
| Smartphone | $\delta_1$ Smartphone bl → BA fu | **.116** | [.021, .211] | .017$^{M}$ | -.018 | [-.053, .017] | .303 | **.132** | [.096, .169] | <.001$^{L}$ | .038 | [-.006, .082] | .087$^{S}$ | | | | | | |
| | $\delta_2$ BA bl → Smartphone fu | .021 | [-.062, .104] | .620 | .022 | [-.011, .054] | .189 | **.130** | [.095, .165] | <.001$^{L}$ | **.056** | [.016, .096] | .006$^{S}$ | | | | | | |
| Work | $\delta_1$ Work bl → BA fu | -.019 | [-.105, .066] | .661 | .002 | [-.029, .034] | .891 | **.095** | [.066, .123] | <.001$^{M}$ | .034 | [-.007, .074] | .108$^{S}$ | **.040** | [.010, .070] | .009$^{S}$ | | | |
| | $\delta_2$ BA bl → Work fu | .061 | [-.011, .133] | .097$^{S}$ | .022 | [-.010, .054] | .186 | **.039** | [.010, .069] | .009$^{S}$ | .037 | [-.003, .078] | .073$^{S}$ | **.055** | [.024, .085] | <.001$^{S}$ | | | |
| **Substance use disorders** | | | | | | | | | | | | | | | | | | | |
| AUD | $\delta_1$ AUD bl → BA fu | .114 | [-.023, .252] | .103$^{M}$ | .013 | [-.029, .055] | .536 | **.052** | [.011, .093] | .014$^{S}$ | .016 | [-.052, .084] | .643 | **.053** | [.013, .093] | .009$^{S}$ | .021 | [-.018, .061] | .294 |
| | $\delta_2$ BA bl → AUD fu | -.032 | [-.153, .088] | .601$^{S}$ | -.013 | [-.057, .030] | .547 | .022 | [-.020, .063] | .306 | .019 | [-.036, .074] | .501 | -.001 | [-.043, .040] | .947 | -.003 | [-.044, .038] | .892 |
| CUD | $\delta_1$ CUD bl → BA fu | .071 | [-.057, .200] | .277$^{M}$ | **.070** | [.023, .116] | **.003**$^{M}$ | .009 | [-.032, .050] | .659 | .028 | [-.027, .083] | .321 | .001 | [-.039, .042] | .945 | .021 | [-.015, .058] | .257 |
| | $\delta_2$ BA bl → CUD fu | -.031 | [-.131, .070] | .552$^{S}$ | -.020 | [-.060, .020] | .334 | -.015 | [-.049, .020] | .407 | -.043 | [-.094, .009] | .104$^{S}$ | -.013 | [-.047, .021] | .454 | **-.037** | [-.072, -.001] | .042$^{S}$ |
| **Mental health problems** | | | | | | | | | | | | | | | | | | | |
| MDD | $\delta_1$ MDD bl → BA fu | .025 | [-.066, .116] | .591 | **.060** | [.026, .094] | .001$^{S}$ | **.080** | [.049, .111] | <.001$^{M}$ | **.059** | [.017, .101] | .006$^{S}$ | **.068** | [.039, .098] | <.001$^{S}$ | **.077** | [.044, .110] | <.001$^{M}$ |
| | $\delta_2$ BA bl → MDD fu | **.086** | [.008, .164] | .030$^{M}$ | **.073** | [.040, .107] | <.001$^{M}$ | **.116** | [.084, .147] | <.001$^{M}$ | **.103** | [.061, .145] | <.001$^{M}$ | **.055** | [.025, .086] | <.001$^{S}$ | **.097** | [.064, .131] | <.001$^{M}$ |
| ADHD | $\delta_1$ ADHD bl → BA fu | -.040 | [-.135, .055] | .407$^{S}$ | **.064** | [.028, .100] | <.001$^{S}$ | **.086** | [.053, .120] | <.001$^{M}$ | **.074** | [.027, .121] | .002$^{M}$ | **.066** | [.033, .098] | <.001$^{S}$ | -.015 | [-.046, .016] | .340 |
| | $\delta_2$ BA bl → ADHD fu | .000 | [-.079, .079] | .996 | **.087** | [.052, .122] | <.001$^{M}$ | **.180** | [.146, .213] | <.001$^{L}$ | **.097** | [.053, .140] | <.001$^{M}$ | **.107** | [.074, .140] | <.001$^{M}$ | **.095** | [.063, .127] | <.001$^{M}$ |
| BPD | $\delta_1$ BPD bl → BA fu | .046 | [-.054, .146] | .370$^{S}$ | **.071** | [.032, .110] | <.001$^{M}$ | **.066** | [.030, .103] | <.001$^{S}$ | .029 | [-.022, .080] | .261 | **.041** | [.007, .076] | .018$^{S}$ | **.080** | [.043, .116] | <.001$^{M}$ |
| | $\delta_2$ BA bl → BPD fu | .047 | [-.059, .152] | .387$^{S}$ | **.043** | [.005, .082] | .026$^{S}$ | .025 | [-.010, .061] | .166 | **.065** | [.016, .115] | .010$^{S}$ | -.010 | [-.046, .026] | .578 | .029 | [-.008, .065] | .120 |
| SAD | $\delta_1$ SAD bl → BA fu | .044 | [-.049, .136] | .356$^{S}$ | .001 | [-.034, .037] | .944 | **.067** | [.036, .097] | <.001$^{S}$ | **.042** | [.002, .083] | .039$^{S}$ | **.074** | [.043, .104] | <.001$^{M}$ | **.071** | [.041, .101] | <.001$^{M}$ |
| | $\delta_2$ BA bl → SAD fu | **.089** | [.014, .164] | .019$^{M}$ | .033 | [-.002, .067] | .063$^{S}$ | **.098** | [.066, .129] | <.001$^{M}$ | **.079** | [.040, .119] | <.001$^{M}$ | **.044** | [.012, .076] | .008$^{S}$ | **.089** | [.058, .120] | <.001$^{M}$ |

Note: BAs, SUDs, and MHPs were modeled as continuous latent variables indicating symptom severity. Gambling = gambling disorder; gaming = problematic gaming; internet =problematic internet use; pornography = problematic internet pornography use; smartphone = problematic smartphone use; work = work addiction; AUD = alcohol use disorder; CUD = cannabis use disorder; MDD = major depressive disorder; ADHD = attention-deficit/hyperactivity disorder; BPD = borderline personality disorder; SAD = social anxiety disorder; $\delta_1$ = pathway between mental health problem (BA, SUD, or other MHP) at baseline and behavioral addiction (BA) at follow-up; $\delta_2$ = pathway between behavioral addiction (BA) at baseline and mental health problem (BA, SUD, or other MHP) at follow-up. β = standardized path coefficient; CI = confidence interval.

Full Information Maximum Likelihood (FIML) was used, allowing for the inclusion of participants with missing values under the 'missing at random' assumption (n = 5611). **Bold** font indicates significant coefficients ($p < .05$). $^{S/M/L}$ = small/medium/large effect size according to Orth et al. (2024).

**References**


Adamson, S. J., & Sellman, J. D. (2003). A prototype screening instrument for cannabis use disorder: the Cannabis Use Disorders Identification Test (CUDIT) in an alcohol-dependent clinical sample. *Drug and alcohol review*, *22*(3), 309-315. https://doi.org/10.1080/0959523031000154454

Andreassen, C. S., Griffiths, M. D., Hetland, J., & Pallesen, S. (2012). Development of a work addiction scale. *Scandinavian journal of psychology*, *53*(3), 265-272. https://doi.org/10.1111/j.1467-9450.2012.00947.x

Annaheim, B., Scotto, T. J., & Gmel, G. (2010). Revising the Cannabis Use Disorders Identification Test (CUDIT) by means of item response theory. *International journal of methods in psychiatric research*, *19*(3), 142-155. https://doi.org/10.1002/mpr.308

American Psychiatric Association (APA) (1994). *Diagnostic and statistical manual of mental disorders: DSM-IV (4th ed.)*. The American Psychiatric Association (APA).

American Psychiatric Association (APA) (2013). *Diagnostic and statistical manual of mental disorders: DSM-5™ (5th ed.)*. The American Psychiatric Association (APA).

Bech, P., Rasmussen, N.-A., Olsen, L. R., Noerholm, V., & Abildgaard, W. (2001). The sensitivity and specificity of the Major Depression Inventory, using the Present State Examination as the index of diagnostic validity. *Journal of affective disorders*, *66*(2-3), 159-164. https://doi.org/10.1016/S0165-0327(00)00309-8

Bech, P., Timmerby, N., Martiny, K., Lunde, M., & Søndergaard, S. (2015). Psychometric evaluation of the Major Depression Inventory (MDI) as depression severity scale using the LEAD (Longitudinal Expert Assessment of All Data) as index of validity. *BMC psychiatry*, *15*, 1-7. https://doi.org/10.1186/s12888-015-0529-3

Beck, F. G., & Legleye, S. (2008). Measuring cannabis-related problems and dependence at the population level. In *A cannabis reader: global issues and local experiences. Vol. 2: Perspectives on cannabis controversies, treatment and regulation in Europe* (pp. 29-57).

Carnes, P. J., Delmonico, D. L., & Griffin, E. (2009). *In the shadows of the net: Breaking free of compulsive online sexual behavior*. Simon and Schuster.

Cohen, J. (1988). The concepts of power analysis. In *Statistical power analysis for the behavioral sciences* (Vol. 2, pp. 1-17). Lawrence Erlbaum Associates.

Dalrymple, K., Martinez, J., Tepe, E., Young, D., Chelminski, I., Morgan, T., & Zimmerman, M. (2013). A clinically useful social anxiety disorder outcome scale. *Comprehensive Psychiatry*, *54*(7), 758-765. https://doi.org/10.1016/j.comppsych.2013.02.006

Delmonico, D., & Miller, J. (2003). The Internet Sex Screening Test: A comparison of sexual compulsives versus non-sexual compulsives. *Sexual and Relationship Therapy*, *18*(3), 261-276. https://doi.org/10.1080/1468199031000153900

Edwards, G., Arif, A., & Hadgson, R. (1981). Nomenclature and classification of drug-and alcohol-related problems: a WHO Memorandum. *Bulletin of the World Health Organization*, *59*(2), 225-242.

First, M. B., Spitzer, R. L., Gibbon Miriam, W., & Janet, B. (1997). *Structured clinical interview for DSM-IV axis I disorders: SCID-I: clinical version: administration booklet* ((No Title), Issue.

Grant, B. F., Dawson, D. A., Stinson, F. S., Chou, P. S., Kay, W., & Pickering, R. (2003). The Alcohol Use Disorder and Associated Disabilities Interview Schedule-IV (AUDADIS-IV): reliability of alcohol consumption, tobacco use, family history of depression and psychiatric diagnostic modules in a general population sample. *Drug and alcohol dependence*, *71*(1), 7-16. https://doi.org/10.1016/s0376-8716(03)00070-x

Griffiths, M. (1999). Internet addiction: Fact or fiction? *The psychologist*.

Griffiths, M. (2005). A 'components' model of addiction within a biopsychosocial framework. *Journal of Substance use*, *10*(4), 191-197. https://doi.org/10.1080/14659890500114359

Griffiths, M., & Davies, M. (2005). *Does video game addiction exist* (Vol. 359).

Guertler, D., Broda, A., Bischof, A., Kastirke, N., Meerkerk, G.-J., John, U., Meyer, C., & Rumpf, H.-J. (2014). Factor structure of the compulsive internet use scale. *Cyberpsychology, Behavior, and Social Networking*, *17*(1), 46-51.

Haug, S., Castro, R. P., Kwon, M., Filler, A., Kowatsch, T., & Schaub, M. P. (2015). Smartphone use and smartphone addiction among young people in Switzerland. *Journal of behavioral addictions*, *4*(4), 299-307. https://doi.org/10.1556/2006.4.2015.037

Kessler, R. C., Adler, L., Ames, M., Demler, O., Faraone, S., Hiripi, E., Howes, M. J., Jin, R., Secnik, K., & Spencer, T. (2005). The World Health Organization Adult ADHD Self-Report Scale (ASRS): a short screening scale for use in the general population. *Psychological Medicine*, *35*(2), 245-256. https://doi.org/10.1017/s0033291704002892

Khazaal, Y., Chatton, A., Horn, A., Achab, S., Thorens, G., Zullino, D., & Billieux, J. (2012). French validation of the compulsive internet use scale (CIUS). *Psychiatric Quarterly*, *83*, 397-405.

Khazaal, Y., Chatton, A., Rothen, S., Achab, S., Thorens, G., Zullino, D., & Gmel, G. (2016). Psychometric properties of the 7-item game addiction scale among french and German speaking adults. *BMC psychiatry*, *16*, 1-10. https://doi.org/10.1186/s12888-016-0836-3

Knight, J. R., Wechsler, H., Kuo, M., Seibring, M., Weitzman, E. R., & Schuckit, M. A. (2002). Alcohol abuse and dependence among US college students. *Journal of studies on alcohol*, *63*(3), 263-270. https://doi.org/10.15288/jsa.2002.63.263

Kwon, M., Kim, D.-J., Cho, H., & Yang, S. (2013). The smartphone addiction scale: development and validation of a short version for adolescents. *PLoS one*, *8*(12), e83558. https://doi.org/10.1371/journal.pone.0083558

Kwon, M., Lee, J.-Y., Won, W.-Y., Park, J.-W., Min, J.-A., Hahn, C., Gu, X., Choi, J.-H., & Kim, D.-J. (2013). Development and validation of a smartphone addiction scale (SAS). *PLoS one*, *8*(2), e56936. https://doi.org/10.1371/journal.pone.0056936

Lemmens, J. S., Valkenburg, P. M., & Peter, J. (2009). Development and validation of a game addiction scale for adolescents. *Media psychology*, *12*(1), 77-95. https://doi.org/10.1080/15213260802669458

Meerkerk, G.-J., van den Eijnden, R. J., Franken, I. H., & Garretsen, H. (2010). Is compulsive internet use related to sensitivity to reward and punishment, and impulsivity? *Computers in Human Behavior*, *26*(4), 729-735. https://doi.org/10.1016/j.chb.2010.01.009

Meerkerk, G.-J., Van Den Eijnden, R. J., Vermulst, A. A., & Garretsen, H. F. (2009). The compulsive internet use scale (CIUS): some psychometric properties. *Cyberpsychology & behavior*, *12*(1), 1-6. https://doi.org/10.1089/cpb.2008.0181

Melartin, T., Häkkinen, M., Koivisto, M., Suominen, K., & Isometsä, E. (2009). Screening of psychiatric outpatients for borderline personality disorder with the McLean Screening Instrument for Borderline Personality Disorder (MSI-BPD). *Nordic Journal of Psychiatry*, *63*(6), 475-479. https://doi.org/10.3109/08039480903062968

Office of Alcoholism and Substance Abuse Services. (2011). *DSM-IV Pathological Gambling Diagnostic Form*. Office of Alcoholism and Substance Abuse Services (OASAS).

Olsen, L. R., Jensen, D., Noerholm, V., Martiny, K., & Bech, P. (2003). The internal and external validity of the Major Depression Inventory in measuring severity of depressive states. *Psychological Medicine*, *33*(2), 351-356. https://doi.org/10.1017/s0033291702006724.

Orth, U., Meier, L. L., Bühler, J. L., Dapp, L. C., Krauss, S., Messerli, D., & Robins, R. W. (2024). Effect size guidelines for cross-lagged effects. *Psychological methods*, *29*(2), 421–433. https://doi.org/10.1037/met0000499

Peterson, R. A., & Brown, S. P. (2005). On the use of beta coefficients in meta-analysis. *Journal of Applied Psychology*, *90*(1), 175. https://doi.org/10.1037/0021-9010.90.1.175

Saunders, J. B., Aasland, O. G., Babor, T. F., De la Fuente, J. R., & Grant, M. (1993). Development of the alcohol use disorders identification test (AUDIT): WHO collaborative project on early detection of persons with harmful alcohol consumption-II. *Addiction*, *88*(6), 791-804.

World Health Organization. (1992). *International statistical classification of diseases and related health problems: 10th revision (ICD-10)*.

World Health Organization. (2018). *International classification of diseases for mortality and morbidity statistics (11th ed.)*. World Health Organization.

Zanarini, M. C., Vujanovic, A. A., Parachini, E. A., Boulanger, J. L., Frankenburg, F. R., & Hennen, J. (2003). A screening measure for BPD: The McLean screening instrument for borderline personality disorder (MSI-BPD). *Journal of personality disorders*, *17*(6), 568-573. https://doi.org/10.1521/pedi.17.6.568.25355

Zimmerman, M., & Mattia, J. I. (2001). A self-report scale to help make psychiatric diagnoses: the Psychiatric Diagnostic Screening Questionnaire. *Archives of general psychiatry*, *58*(8), 787-794. https://doi.org/10.1001/archpsyc.58.8.787
